# Transforming atmospheric CO_2_ into alternative fuels: a metal-free approach under ambient conditions†
†Electronic supplementary information (ESI) available: Experimental section, NMR data, a video clip, computational details, XRD details with CIF files and NMR images. CCDC 1583077–1583080. For ESI and crystallographic data in CIF or other electronic format see DOI: 10.1039/c8sc03581d


**DOI:** 10.1039/c8sc03581d

**Published:** 2018-11-30

**Authors:** Samaresh Chandra Sau, Rameswar Bhattacharjee, Pradip Kumar Hota, Pavan K. Vardhanapu, Gonela Vijaykumar, R. Govindarajan, Ayan Datta, Swadhin K. Mandal

**Affiliations:** a School of Chemical Sciences , Indian Association for the Cultivation of Science , 2A and 2B Raja S. C. Mullick Road, Jadavpur 700032 , Kolkata , West Bengal , India . Email: spad@iacs.res.in; b Department of Chemical Sciences , Indian Institute of Science Education and Research Kolkata , Mohanpur 741246, Nadia , West Bengal , India . Email: swadhin.mandal@iiserkol.ac.in

## Abstract

This work demonstrates the first-ever completely metal-free approach to the capture of CO_2_ from air followed by reduction to methoxyborane (which produces methanol on hydrolysis) or sodium formate (which produces formic acid on hydrolysis) under ambient conditions. This was accomplished using an abnormal N-heterocyclic carbene (*a*NHC)–borane adduct. The intermediate involved in CO_2_ capture (*a*NHC-H, HCOO, B(OH)_3_) was structurally characterized by single-crystal X-ray diffraction. Interestingly, the captured CO_2_ can be released by heating the intermediate, or by passing this compound through an ion-exchange resin. The capture of CO_2_ from air can even proceed in the solid state *via* the formation of a bicarbonate complex (*a*NHC-H, HCO_3_, B(OH)_3_), which was also structurally characterized. A detailed mechanism for this process is proposed based on tandem density functional theory calculations and experiments.

## Abstract 2

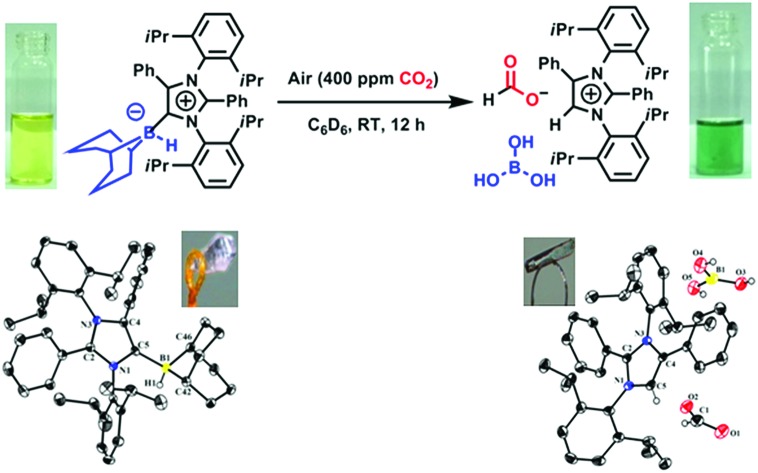
The present work established the metal-free approach to capture CO_2_ from air and its reduction into alternative fuel under ambient conditions.

## Introduction

**Fig. 1:**
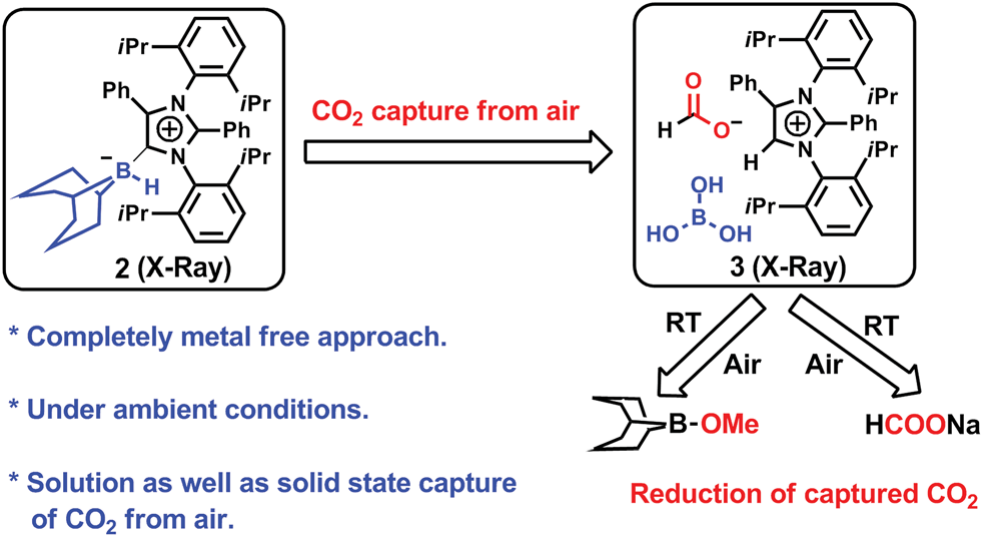
Metal-free CO_2_ fixation from air followed by reduction to methoxyborane or sodium formate under ambient conditions.

Carbon dioxide is an attractive, economical, and renewable C_1_ source for the production of value added chemicals and fuels.^1^–^5^ Many fuel-related C_1_ products, such as methane (CH_4_), methanol (CH_3_OH) and formic acid (HCOOH), can be obtained by treating CO_2_ with a reducing agent as a hydride source.^6^ Among the various possible CO_2_ hydrogenation products, CH_3_OH and formic acid are the most attractive candidates. Methanol can be used as a direct replacement for gasoline in internal combustion engines and also in direct methanol fuel cells.^7^ In addition, formic acid has applications in the textile industry in dyeing processes, as a food preservative and as a hydrogen storage material.^8^–^10^ During the last 20 years, numerous reports have described the reduction of CO_2_ by various transition metal based compounds.^11^–^17^ The reduction was also accomplished catalytically without a transition metal based compound^18^ or by direct reduction with NaBH_4_ without requiring any catalyst.^19^ The reduction of CO_2_ has been achieved even with metal-free catalysts.^20^–^25^ These reports utilized commercially available pure CO_2_ gas from a cylinder. However, it remains difficult to capture CO_2_ present in the ambient atmosphere at extremely low concentrations and to perform reduction reactions to obtain fuels under ambient conditions. Only a very few compounds^26^–^32^ have demonstrated selective CO_2_ absorption from air, but these compounds are not capable of reducing the captured CO_2_ to generate methanol. Recently, Olah and coworkers combined two compounds with the aim of capturing CO_2_ from air followed by reduction into methanol. However, this process required the use of a rare and expensive Ru-based species at 155 °C under harsh reaction conditions (50 bar H_2_ pressure).^33^ At present, there are no methods for the capture of CO_2_ from the atmosphere and the consecutive reduction of this CO_2_ to produce methoxyborane (which can be hydrolyzed to methanol) under ambient conditions using a single chemical system without any metal-based compounds. Moreover, there is currently only a very limited understanding of the mechanistic pathways for such key chemical transformations based on isolating and characterizing various intermediates. Density functional theory (DFT) calculations along with several analyses, including structure determination by single-crystal X-ray diffraction (SC-XRD), were employed in this work to gain better insights into the mechanistic details of the conversion. The present study describes metal-free capture of CO_2_ from air and its reduction under ambient conditions (Fig. 1) using an abnormal N-heterocyclic carbene.

**Fig. 2:**
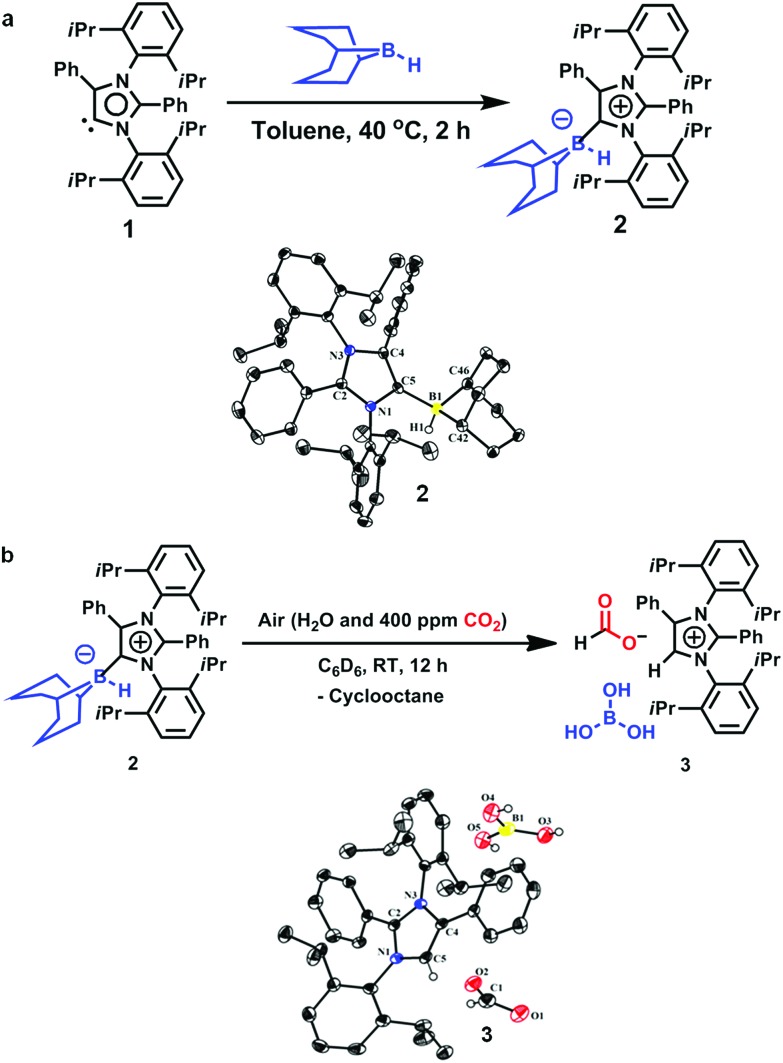
Fixation of CO_2_ in air. (a) Synthesis of an abnormal N-heterocyclic carbene–9-borabicyclo(3.3.1)nonane adduct (*a*NHC–9BBN, **2**) and an ORTEP drawing of the molecular structure of **2**. (b) Fixation of CO_2_ from air with the formation of **3** and an ORTEP drawing of **3**.

An abnormal N-heterocyclic carbene (*a*NHC; **1**) was prepared according to a literature procedure,^34^ following which the *a*NHC–9BBN adduct, **2**, was synthesized in a 75% isolated yield by reacting **1** and 9-BBN in a 1 : 1 equivalent ratio in toluene at 40 °C for 2 h (Fig. 2a). Because a higher yield and crystals suitable for XRD analysis were obtained, this reaction protocol represents an improvement over the previously reported procedure by Crudden and coworkers using an *a*NHC salt, in which the XRD structural analysis of the adduct **2** was not carried out.^35^ Crystals of **2** suitable for XRD analysis were successfully grown from a toluene/hexane mixture under an inert atmosphere and the resulting structure is presented in Fig. 2a. When a solution of **2** in deuterated benzene was left exposed to the ambient air overnight, a distinct color change from light yellow to green was observed. The formation of a new product (**3**, Fig. 2b) was also confirmed by ^1^H NMR spectroscopy. The conversion of the original compound during this process was 66% and the isolated crystal yield of **3** was 40%. The ^1^H NMR spectrum of **3** in deuterated chloroform showed two singlets at *δ* = 8.53 and 8.55 ppm with a 1 : 1 signal intensity ratio, attributed to the formate anion^36^ and C5–H of the azolium cation,^37^ respectively. The presence of formate anions indicated that CO_2_ in the ambient air may have been fixed during the transformation to provide these species. The ^13^C NMR spectrum of **3** contains signals at *δ* = 169.2 ppm (assigned to the C=O of formate) and 125.4 ppm (the C5 of azolium), which further substantiates the incorporation of a formate anion in **3**.^24^,^36^ The fate of the B atom in the 9-BBN backbone of **3** during this transformation was determined using ^11^B NMR spectroscopy, which showed a singlet at *δ* = 20.8 ppm, attributed to free boric acid {B(OH)_3_}. Based on these data, a structure for **3** was developed (Fig. 2b).This compound was successfully crystallized into colorless crystals from a benzene/*n*-hexane mixture in open atmosphere. Single-crystal XRD analysis confirmed the proposed structure (Fig. 2b), in which a CO_2_ molecule is fixed as a formate anion by reaction with the *a*NHC–borane adduct **2**.

**Scheme 1:**
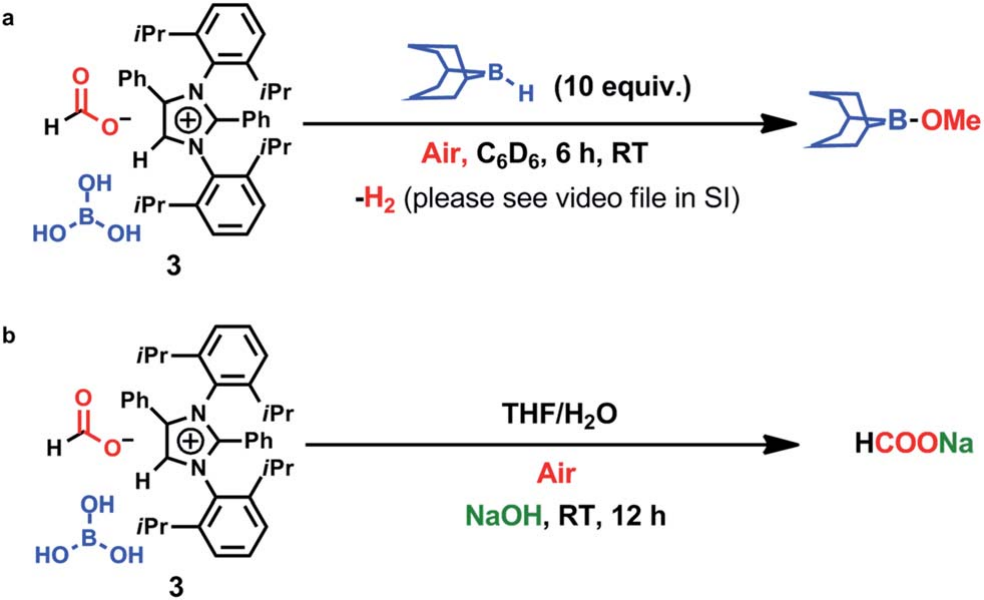
Reduction of CO_2_ to methoxyborane and sodium formate in air.

**Scheme 2:**
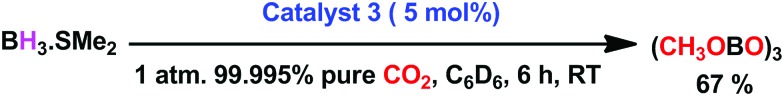
Metal-free catalytic reduction of CO_2_ under ambient conditions.

**Fig. 3:**
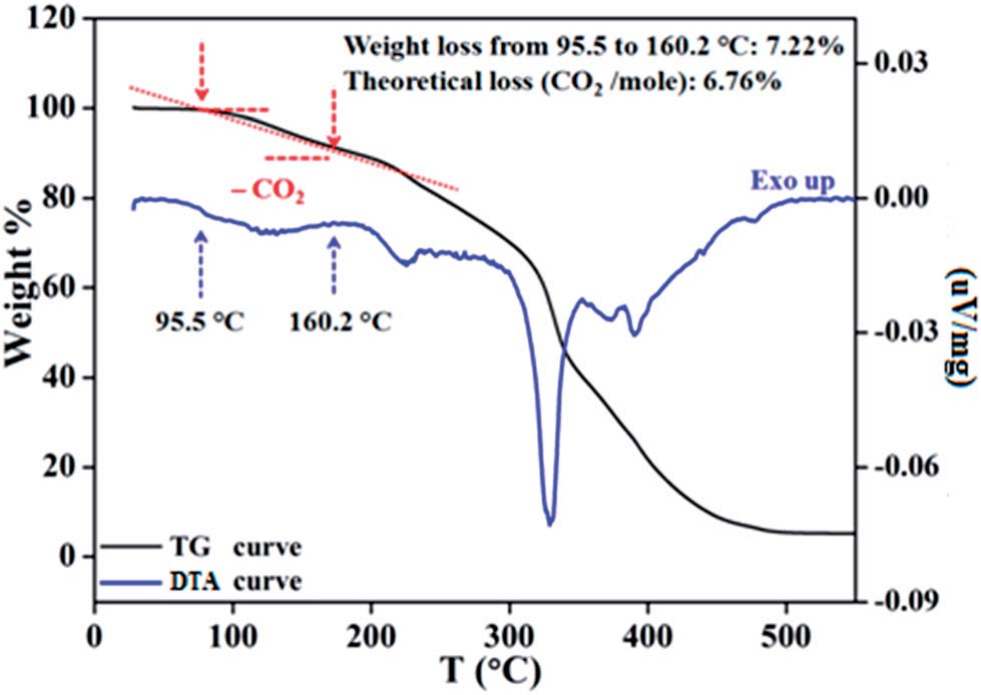
Removal of captured CO_2_ in solid state, as monitored by thermogravimetric-differential thermal analysis of compound **3**.

**Fig. 4:**
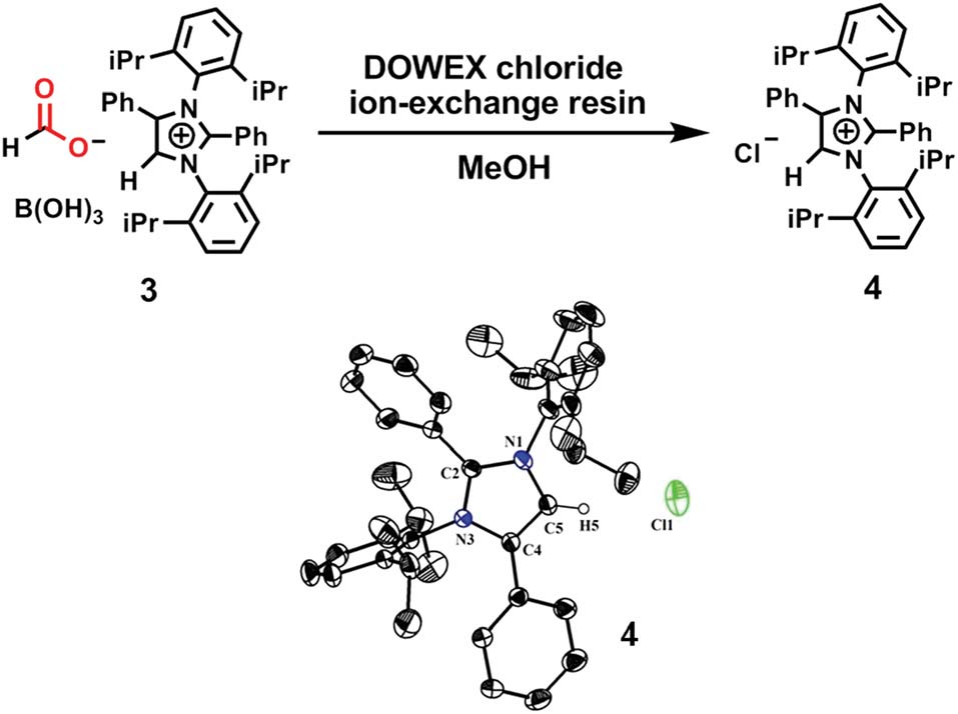
Substitution of a formate anion with a chloride ion upon passing **3** through an ion-exchange resin, and an ORTEP drawing of the molecular structure of **4**.

**Fig. 5:**
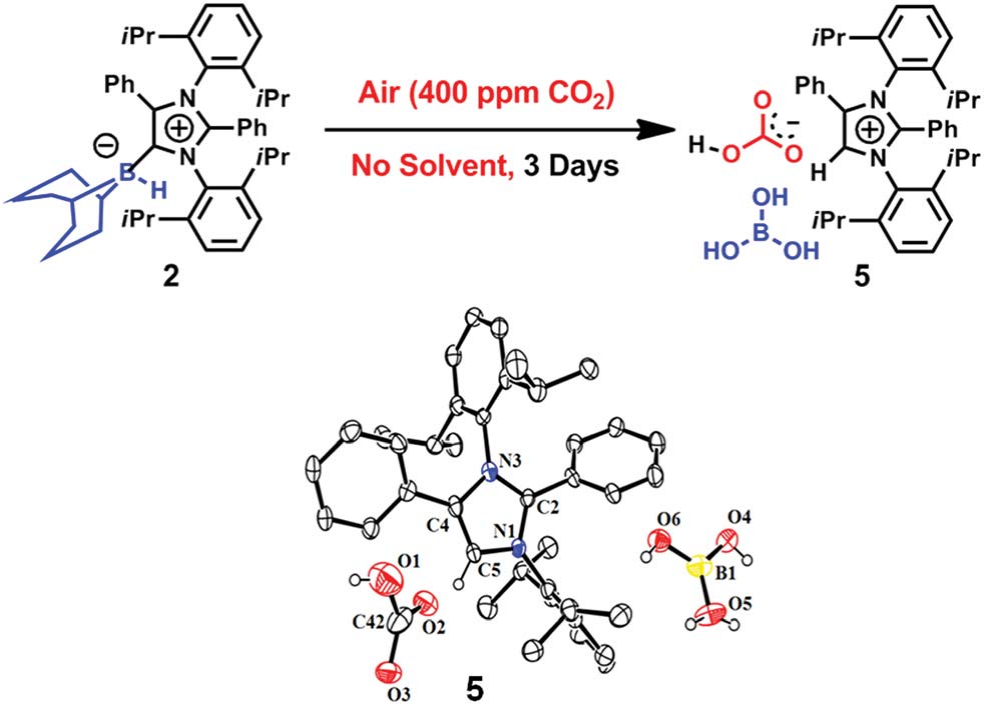
Capture of CO_2_ from air by **2** in the solid state with the formation of the bicarbonate **5**, and its ORTEP drawing.

To ascertain the reactivity of the captured CO_2_, we treated **3** with a boron hydride. Upon reaction of **3** with 10 equiv. of the hydroborane 9-BBN in the presence of air, the corresponding methoxyborane product (CH_3_OBBN) was obtained with complete consumption of **3** within 6 h in deuterated benzene (Scheme 1a, methoxyborane). The formation of CH_3_OBBN was confirmed by ^1^H NMR (*δ* = 3.44 ppm) and ^11^B NMR (*δ* = 57 ppm) spectroscopy in deuterated benzene and by comparing our results to previous literature data.^20^ During the course of the reaction, gas evolution was observed (see the video file in the ESI†), which was confirmed to be H_2_ by ^1^H NMR spectroscopy (*δ* = 4.47 ppm).^38^ Additionally, upon introducing dry CO_2_ from a cylinder (99.995% pure) and performing the same reaction under moisture-free pure CO_2_ instead of air, **3** exhibited catalytic activity, which may be explained based on a mechanism previously proposed by our group.^24^ Under these conditions, CO_2_ was catalytically converted to the corresponding trimethoxyboroxine^21^,^39^ with 67% conversion, using a low cost borane (BH_3_·SMe_2_) as the hydride source (Scheme 2). The feasibility of applying **3** to the synthesis of formic acid *via* the formation of sodium formate was also assessed. Adding **3** to 5 mL of a 2 M sodium hydroxide solution and 0.5 mL tetrahydrofuran at room temperature in air resulted in complete consumption of the compound (Scheme 1b, sodium formate). The formation of sodium formate (HCOONa) was confirmed by ^1^H NMR spectroscopy (*δ* = 8.38 ppm in deuterated water) based on previous literature data.^11^ We subsequently attempted to remove the CO_2_ captured in **3** by heating. Raising the temperature of **3** to 150 °C for 12 h in the solid state was found to promote the loss of the formate anion, leading to an abrupt color change from colorless to brown, and forming a new product as determined by ^1^H NMR spectroscopy.^40^,^41^ As noted previously, **3** showed two singlets in its ^1^H NMR spectrum at *δ* = 8.53 and 8.55 ppm with a 1 : 1 intensity ratio in deuterated chloroform. After heating **3** at 150 °C for 12 h, the ^1^H NMR spectrum of the resulting compound contained only a new singlet (*δ* = 8.35 ppm) while the other original singlet was absent. This observation indicates that the formate anion might have been lost during the heating process. To further support this assumption, a ^13^C NMR spectroscopic experiment was performed. Following heating, the singlet in the downfield region of the ^13^C NMR spectrum acquired in deuterated chloroform (at *δ* = 169.2 ppm) disappeared completely, confirming the loss of the formate counter anion. Thermogravimetric-differential thermal analysis (TG-DTA) analyses were also performed by heating **3** from 27 to 300 °C at a rate of 4 °C min^–1^. The TG data in Fig. 3 (black line) demonstrate mass loss beginning at 95.5 °C and continuing up to 160.2 °C, for a total loss of 7.22% (going from a molecular mass of 651.4 to 603.9). This may be attributed to the loss of a CO_2_ molecule from **3** on heating (the theoretical mass loss is 6.76%). The DTA data in Fig. 3 (blue line) exhibit an exotherm in agreement with the loss of CO_2_. Based on the NMR evidence and the TG-DTA analysis,^30^,^42^–^44^ we may conclude that heating **3** at 150 °C releases the captured CO_2_ that is present as a formate anion. It was also found possible to replace the formate anion in **3** with a chloride ion simply by passing it through a Dowex ion-exchange resin. This procedure generated compound **4** with a 50% isolated yield (Fig. 4).^45^ The formation of **4** was confirmed by NMR analysis and single-crystal XRD. The ^1^H NMR spectrum of the resulting compound in deuterated chloroform contained only one singlet at *δ* = 8.84 ppm, while the ^13^C NMR spectrum did not display any signals beyond 145.1 ppm, confirming the absence of formate ions in **4**. Finally, **4** was obtained as colorless crystals from methanol, and single-crystal XRD confirmed the expected structure (Fig. 4), in which the formate anion was replaced by a chloride ion.^45^ Based on the above ion exchange chromatographic experiment, it was possible to regenerate the *a*NHC salt (**4**). Interestingly, when **2** was exposed to air in the solid state for three days as a fine powder in a Petri dish, the formation of a new product was observed, as evidenced by ^1^H NMR spectroscopy (Fig. 5).^26^,^30^,^46^

The ^1^H NMR spectrum of this new compound in deuterated chloroform at room temperature contained two singlets at *δ* = 8.58 and 8.77 ppm with a 1 : 1 intensity ratio. These two signals (which are different from those produced by **3**) were attributed to a bicarbonate anion^30^ and an imidazolium cation,^24^ respectively. These assignments were further substantiated by the corresponding ^13^C NMR spectroscopic signals at *δ* = 169 ppm (C=O of bicarbonate) and 125.4 ppm (C5 of azolium). Furthermore, the ^11^B NMR spectrum of **5** exhibits a singlet at *δ* = 21 ppm that can be ascribed to free boric acid. Based on these data, it is evident that **5** was obtained (Fig. 5). Colorless crystals of **5** were grown from a benzene/*n*-hexane mixture with a 50% yield and the single-crystal XRD analysis of compound **5** confirmed the expected structure (Fig. 5).

**Fig. 6:**
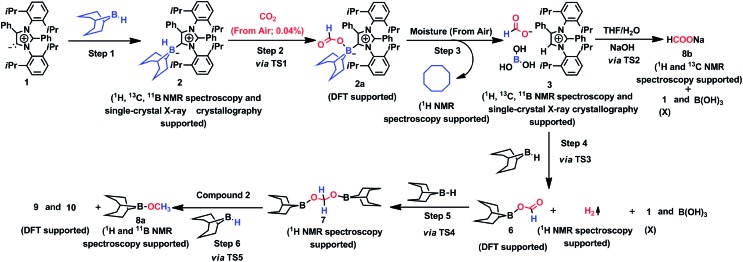
Mechanistic scheme for the reduction of CO_2_ to methoxyborane and sodium formate with **2** in air. For drawings of **9** and **10** see Fig. S3.†

**Fig. 7:**
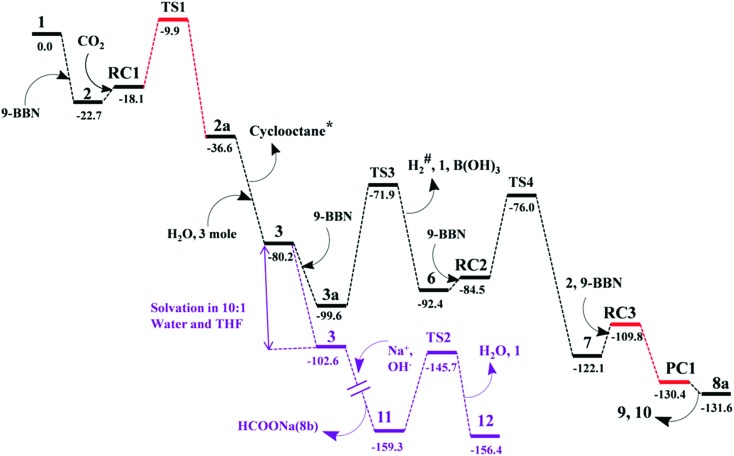
Computed Gibbs free energy profiles at 25 °C for the conversion of CO_2_ to methoxyborane or sodium formate with **2** in air. The relative free energies (in kcal mol^–1^) obtained in solvent were calculated with respect to the energy of the separate reactants {*a*NHC (**1**) and 9-BBN}. *Formation of cyclooctane was confirmed by ^1^H NMR spectroscopy. ^#^H_2_ gas evolution was confirmed by ^1^H NMR spectroscopy and visual observation (see the video file in the ESI†). For drawings of all compounds see Fig. S3.†

**Fig. 8:**
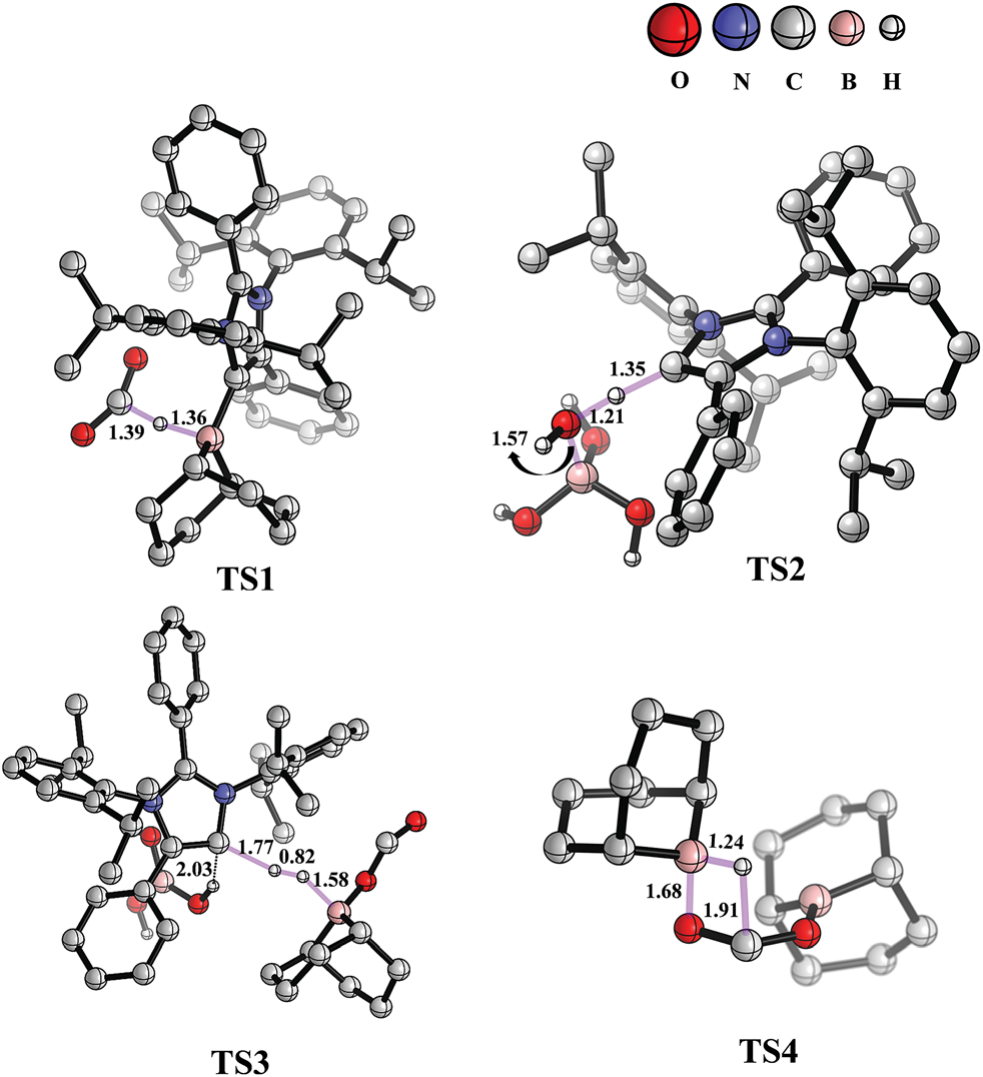
Optimized structures of the transition states involved in the potential energy surface of CO_2_ reduction to methoxyborane and sodium formate with **2** in air. Important bond distances (in Å) are shown. Only relevant H atoms are provided in the structures.

The mechanism associated with the reduction of the captured CO_2_ was examined by performing several stoichiometric reactions in addition to high level DFT calculations. On the basis of the experimental results, a full mechanistic path (Fig. 6) is proposed together with the energy profile diagram shown in Fig. 7. The optimized transition states for the mechanistic path are presented in Fig. 8. The combination of **1** with 9-BBN in an equimolar amount in toluene at 40 °C furnishes **2**,^35^ as confirmed by NMR and XRD analysis. The electrostatic potential (ESP) surface and the highest occupied molecular orbital of **2** were also determined (Fig. S1, ESI†). In the ESP surface diagram, the red region around the B–H bond of intermediate **2** indicates higher electron density around that area. Thus, **2** could potentially serve as a hydride donor during the course of the reaction. This can be explained by the activation of the B–H bond of **2** as a result of its proximity to the strongly σ-donating *a*NHC moiety. In the presence of air, a CO_2_ molecule is selectively incorporated into the B–H bond^36^,^47^ of **2** to form **2a***via* reactant complex **RC1** (Fig. S3†) and transition state **TS1** (Fig. 8). During the reaction of **2** with dry air, we observed the capture of CO_2_ as formate ions^36^ as confirmed by the corresponding ^13^C NMR signal at *δ* = 168.2 ppm (Fig. S25†). The activation barrier for this elementary step is much lower (Δ*G*^‡^ = 8.2 kcal mol^–1^). The formation of **2a** is thermodynamically favorable, as this particular step involves a heat release of 18.5 kcal mol^–1^. As a result of atmospheric moisture, **2a** is hydrolyzed to **3**, which stabilizes the compound as a zwitterion.^47^ During this process, the 9-BBN fragment is hydrolyzed to boric acid with the elimination of a cyclooctane molecule, as confirmed by ^1^H NMR analysis (*δ* = 1.55 ppm in deuterated benzene) of the reaction mixture.^48^ Compound **3** was thoroughly characterized by XRD, NMR spectroscopy and elemental analysis. This compound was also found to react with sodium hydroxide solution to furnish sodium formate (**8b**), as confirmed through NMR analysis.^11^

When sodium hydroxide was added to a solution of **3** in water/tetrahydrofuran, sodium formate was released with the formation of intermediate **11** (Fig. S3†). The structure of **11**, as shown in Fig. S3,† consists of two fragments (*viz*. B(OH)_4_^–^ and *a*NHC-H^+^) that subsequently release a water molecule to form boronic acid (**12**) and free *a*NHC (**1**) (Fig. S3†) *via***TS2** (Fig. 8). The free energy of activation for water elimination was calculated to be 13.6 kcal mol^–1^. In addition, another 9-BBN molecule reacts with **3** to furnish **3a** (Fig. S3†), thus regenerating free *a*NHC (**1**) with the formation of boron formate^20^ (**6**) *via* hydrogen gas evolution^38^ (see the video file in the ESI†) through **TS3** (Fig. 8). The spatial orientation of **TS3** is shown in Fig. 8, in which the distance between the two hydrogen atoms is 0.82 Å. The free energy barrier for this hydrogen evolution reaction was calculated to be 27.7 kcal mol^–1^. In addition, **6** can be reduced to its acetal form (H_2_C(OBBN)_2_; **7**) in the presence of 9-BBN *via***TS4** (Fig. 8), through reactant complex **RC2** (Fig. S3†).^20^,^22^ The free energy barrier for this hydroboration is 8.5 kcal mol^–1^ and this particular step is highly exothermic (Δ*G* = – 37.6 kcal mol^–1^). The formation of **7** was confirmed from its characteristic chemical shift (*δ* = 5.34 ppm in deuterated benzene) upon analysis of the reaction mixture by ^1^H NMR spectroscopy.^20^ Finally, **7** is reduced to the methoxide derivative **8a** in the presence of **2** and 9-BBN (Fig. 7), through reactant complex **RC3** (Fig. S3†) and product complex **PC1** (Fig. S2†).^20^ The formation of **8a** is associated with the elimination of **9** and **10** (Fig. S3†), and the free energy change (Δ*G*) for this exothermic elementary step was determined to be 20.6 kcal mol^–1^.

In summary, in this work we synthesized an abnormal N-heterocyclic carbene (*a*NHC) supported 9-BBN adduct (**2**) for CO_2_ capture from air with subsequent conversion to methoxyborane or sodium formate under ambient conditions. The intermediate (*a*NHC-H, HCOO, B(OH)_3_; **3**) was structurally characterized to unequivocally establish the capture of atmospheric CO_2_. It was further shown that CO_2_ present in low concentrations (such as in ambient air) could be captured by this compound in the solid state. This represents the first-ever demonstration of the possibility of CO_2_ capture from air followed by its reduction to methanol without employing any metal under very mild conditions. A detailed mechanistic pathway for this fascinating transformation was proposed based on tandem experimental and computational studies.

## Conflicts of interest

There are no conflicts to declare.

## Supplementary Material

Supplementary movie

Supplementary information

Crystal structure data
