# Supplementary material for: Transforming atmospheric CO2 into alternative fuels: a metal-free approach under ambient conditions
Source: Chem Sci. 2018 Nov 30;10(6):1879–84. doi: 10.1039/c8sc03581d (PMC6371756; doi:10.1039/c8sc03581d)
Supplement: Supplementary file 2 [file SC-010-C8SC03581D-s002.pdf]

Electronic Supplementary Material (ESI) for Chemical Science.
This journal is © The Royal Society of Chemistry 2018

Transforming Atmospheric CO_2 into Alternative Fuels: a Metal-Free Approach under Ambient Conditions

Samaresh Chandra Sau,† Rameswar Bhattacharjee,‡ Pradip Kumar Hota,† Pavan K. Vardhanapu,† Gonela Vijaykumar,† R. Govindarajan,† Ayan Datta*‡ and Swadhin K. Mandal*†

‡School of Chemical Sciences, Indian Association for the Cultivation of Science, 2A and 2B Raja S. C. Mullick Road, Jadavpur 700032, Kolkata, West Bengal, India. Email: spad@iacs.res.in

†Department of Chemical Sciences, Indian Institute of Science Education and Research Kolkata, Mohanpur 741246, Nadia, West Bengal, India. E-mail: swadhin.mandal@iiserkol.ac.in

Table of Contents

Experimental Section…………………………………………………………………………………………..……..
Experimental Materials and Instrumentation……………………………………………..………………

Synthesis of 1,3-bis(2,6-diisopropylphenyl)-2,4-diphenylimidazolidine-9-borabicyclo[3.3.1]nonane (**2**) ……………………………………………………………………………..………..
Synthesis of 1,3-bis(2,6-diisopropylphenyl)-2,4-diphenylimidazolidineformate boric acid (**3**)…………………………………………………………………………………………………...…………..
Methoxyborane formation with compound **3**……………………………………………………………..
Formation of sodium formate from compound **3**…………………………………………..……………
Catalytic hydroboration of CO_2 with compound **3**…………………………………………………………
Synthesis of {1,3-bis(2,6-diisopropylphenyl)-2,4-diphenyl-imidazolium (Cl-)} (**4**)………………
Expulsion of carbon dioxide from compound **3** under heating ……………………………………………………………………………………………….………….……….
Synthesis of {1,3-bis(2,6-diisopropylphenyl)-2,4-diphenylimidazolidine bicarbonate boric acid} (**5**) ……………………………………………………………………………………….………..……..
Details of TGA study……………………………………..………………………………………………

Computational details…………………………………………………………………..…………………………
Fate of intermediate **10** in presence of moisture………………………………...………………………
Details for preparation of video clipping of the carbon dioxide reduction from air with Compound**3**…………………………………………………………………………..……………….…………….
X-ray crystal structure measurements…………………………..………………………………………
Table S1. Crystallographic and data collection parameters for compounds **2-5**………..………..

Table S2. Selected bond distances (Å) and angles (°) for compounds **2-5**…………...…………..

Table S3. Final coordinates and equivalent isotropic displacement parameters of the non-hydrogen atoms for compound **2**…………………………………………..…………………..
Table S4. Hydrogen atom positions and isotropic displacement parameters for compound **2**……………………………………………………..…………………………………..………..
Table S5. Final coordinates and equivalent isotropic displacement parameters

of the non-hydrogen atoms for compound **3**……………………………………………………………………...
Table S6. Hydrogen atom positions and isotropic displacement parameters for
Compound**3**……………………………………………………………………………………………………...
Table S7. Final coordinates and equivalent isotropic displacement parameters
of the non-hydrogen atoms for compound **4**…………………………………………………………………..
Table S8. Hydrogen atom positions and isotropic displacement parameters
for compound **4**……………………………………………………………………………………………………
Table S9. Final coordinates and equivalent isotropic displacement parameters
of the non-hydrogen atoms for compound **5**………………………………………………………………….
Table S10. Hydrogen atom positions and isotropic displacement parameters
for compound **5**…………………………………………………………………………………………………
^{1}H, ^{13}C and ^{11}B NMR spectra..
References……………………………………………………………………………………………………...

Experimental Section

Experimental Materials and Instrumentation

Compounds **1** and **2** were prepared under a dry and oxygen-free atmosphere (argon) using standard Schlenk techniques or inside a glovebox maintained below 0.1 ppm of O_2 and H_2O levels. Glasswares were dried for overnight at 130 °C before use. THF, toluene, hexane and benzene were dried over a sodium/benzophenone mixture and distilled before use. Apart from compounds **1** and **2**, other compounds were prepared in open atmosphere. Carbon dioxide was purchased from Praxair in a 5.5 purity gas cylinder with 99.995% purity. The ^{1}H and ^{13}C NMR spectra were recorded on 400 and 500 MHz NMR spectrometers with residual undeuterated solvent as an internal standard. ^{11}B NMR spectra were obtained by using a Bruker Avance 500 MHz NMR spectrometer. Chemical shifts for ^{11}B NMR spectra were referenced using $Et_2O{\cdot}BF_3$ as an external standard. Chemical shifts (δ) are given in ppm, and J values are given in Hz. Elemental analyses were performed in a Perkin–Elmer 2400, Series II, CHNS/O analyzer. The HRMS was measured in Bruker Daltonics Micro TOF-Q-II with electro spray ionization (ESI). Thermogravimetry was carried out on NETSZCH TGA-DTA system. All solid reagents or substrates were purchased (Sigma-Aldrich, Merck and Spectrochem) and used as received. Unless otherwise noted, liquid chemicals were purchased from commercial suppliers (Sigma-Aldrich, Merck and Spectrochem) and dried over molecular sieves (4 Å) prior to use. The molecular sieves (4 Å, Merck) were dried under a dynamic vacuum at 250 °C for 48 h prior to use. Isolated abnormal N-heterocyclic carbene (*a*NHC), **1** was prepared according to the literature procedure.[1] Capture of carbon dioxide from air and its reduction into methoxyborane or sodium formate was performed in open atmosphere using a glass vial. During catalysis, we performed freeze-pump-thaw

cycle using J. Young tube and we waited until the temperature of J. Young tube reached to room temperature. Then the reaction mixture was exposed to atmospheric pressure of CO_2 gas and the J. Young tube was sealed to perform the reaction.

Synthesis of 1,3-bis(2,6-diisopropylphenyl)-2,4-diphenylimidazolidine-9-borabicyclo[3.3.1]nonane (2)

A 25 mL Schlenk flask equipped with a stirring bar was charged with **1** (272.0 mg, 0.50 mmol, 1equiv), 9-BBN (0.25 mmol, 1 equiv) and toluene (10 mL) at 40 ^{o}C for 2 h. During this period, reaction mixture becomes transparent from initial green color. After 2 h, the solvent was dried under reduced pressure and the final off white solid product was washed with hexane for three times to afford the title compound **2** (255 mg, 0.375 mmol, 75%). This reaction protocol represents an improvement over the previously reported procedure by Crudden and co-workers using *a*NHC salt.[2] However the X-ray structure of adduct **2** was not known previously. In this study X-ray quality crystals of compound **2** was successfully grown from toluene/hexane mixture under inert atmosphere. ^{1}H NMR (500 MHz, C_6D_6, 25 ^{o}C, TMS): δ = 7.32 (d, J = 7.0 Hz, 2H), 7.29-7.26 (m, 1H), 7.14 (s, 2H), 7.02-6.88 (m, 4H), 6.81-6.78 (m, 4H), 6.55-6.46 (m, 3H), 3.41 (sept, J = 6.5 Hz, 2H), 2.85 (sept, J = 6.5 Hz, 2H), 2.46-2.32 (m, 5H), 2.19-2.17 (m, 2H), 2.06-2.03 (m, 1H), 1.89-1.80 (m, 5H), 1.61 (d, J = 6.5 Hz, 6H), 0.94 (d, J = 7.0 Hz, 6H), 0.77 (d, J = 6.5 Hz, 6H), 0.72 (d, J = 7.0 Hz, 6H) ppm; ^{13}C NMR (125 MHz, C_6D_6, 25 ^{o}C, TMS): δ = 146.1, 145.7, 140.9, 136.7, 135.7, 132.8, 131.9, 131.3, 131.1, 130.5, 129.8, 129.3, 128.8, 127.4, 125.0, 124.9, 124.9, 38.1, 32.5, 29.4, 29.1, 26.9, 26.7, 24.6, 24.1, 23.8, 23.7 ppm; ^{11}B NMR (160 MHz, C_6D_6, 25 ^{o}C, TMS): δ = -15.9 ppm.

Synthesis of 1,3-bis(2,6-diisopropylphenyl)-2,4-diphenylimidazolidineformate boric acid (3)

A 5 mL glass vial was charged with **2** (20.0 mg, 0.029 mmol) with 2 mL benzene in open atmosphere at room temperature for 12 h. During this period we observed a sharp color change from light yellow to green with the evaporation of little amount solvent. The same observation was found in presence of C_6D_6 and toluene also. After 12 h, solvent was dried under reduced pressure and the final product was washed with hexane for three times to afford compound **3**. An X-ray quality crystal of compound **3** (08 mg, 0.012 mmol, 40%) was successfully grown from benzene/*n*-hexane mixture as colorless crystals in open atmosphere.^{1}H NMR (500 MHz, $CDCl_3$, 25 °C, TMS): δ = 8.55 (s, 1H), 8.53 (s, 1H), 7.65-7.57 (m, 2H), 7.44-7.31 (m, 9H), 7.25-7.21 (m, 3H), 6.94 (d, *J* = 7.5 Hz, 2H), 2.54-2.41 (m, 4H), 1.32 (d, *J* = 7.0 Hz, 6H), 1.01 (d, *J* = 6.5 Hz, 6H), 0.87-0.85 (m, 12H) ppm; ^{13}C NMR (125 MHz, $CDCl_3$, 25 °C, TMS): δ = 169.2, 145.3, 144.9, 144.5, 137.2, 132.9, 132.8, 132.5, 130.9, 130.1, 129.6, 129.5, 129.2, 128.4, 126.1, 125.4, 124.3, 123.2, 120.7, 36.4, 29.5, 29.2, 25.4, 23.8, 23.3, 22.5, 20.0 ppm; ^{11}B NMR (160 MHz, $CDCl_3$, 25 °C, TMS): δ = 20.8 ppm. Elemental analysis: Calcd. for $C_{40}H_{52}BN_2O_5$: C 73.72, H 8.04, N 4.30; found: C 71.72, H 7.29, N 4.34.

Methoxyborane formation with compound 3

In a glovebox, borane (0.07 mmol, 10 equiv) was added in a 5 mL glass vial containing compound **3** (5.0 mg, 0.007 mmol) and finally the vial was closed with proper cap and taken outside. To this glass vial, 2 mL C_6D_6 was added after quickly opening the vial in air and then closed with the cap and kept it at RT. Immediately, we observed a gas evolution (please see video clip), which was confirmed as hydrogen gas by ^{1}H NMR spectroscopy (δ =

4.46 ppm). The reaction was continued for 6 h and we observed a sharp color change from light yellow to deep yellow. The formation of methoxyborane with full consumption of starting compound **3** was confirmed by ^{1}H NMR spectroscopy. During this reaction, we observed that 10 equivalents borane were necessary to consume the starting compound **3**. It may be attributed to the fact that some amount of borane might be decomposing on exposure to air before it can react with **3**.

Formation of sodium formate from compound 3

A 15 mL glass vial was charged with **3** (10.0 mg, 0.014 mmol), 5 mL 2(M) NaOH solution and 0.5 mL tetrahydrofuran (THF). The reaction was continued for 12 h by closing the vial with a cap. During this period, we observed a sharp color change of reaction mixture from light yellow to colorless. The formation of sodium formate with full consumption of starting compound **3** was confirmed by ^{1}H NMR spectroscopy using D_2O.

Catalytic hydroboration of CO_2 with compound 3

Under an argon atmosphere, a 25 mL Schlenk tube equipped with a J. Young valve was charged with **3** (5.0 mg, 0.007 mmol), $BH_3.SMe_2$ (0.14 mmol, 20 equiv.) and C_6D_6 (2 mL). To this solution, hexamethylbenzene was added as an internal standard. The mixture was degassed by a freeze-pump-thaw cycle and placed under 1 atm. of CO_2 (99.995% pure CO_2 from a CO_2 cylinder) at room temperature. The progress of the reaction was monitored by ^{1}H NMR spectroscopy and the conversion was calculated based on the integration of methoxy group of $(CH_3OBO)_3$ with respect to internal standard. All reported conversion of product was an average of at least two runs.

Synthesis of {1,3-bis(2,6-diisopropylphenyl)-2,4-diphenyl-imidazolium (Cl-)} (4)

In the open atmosphere, compound **3** (20.0 mg, 0.028 mmol) was passed through a column containing DOWEX chloride ion-exchange resin. The eluent was methanol (MeOH). After column chromatography, the title compound was isolated under reduced pressure as a white solid (08 mg, 0.014 mmol, 50%). Finally compound **4** {1,3-bis(2,6-diisopropylphenyl)-2,4-diphenyl-imidazolium (Cl^-)} was obtained as colorless crystals from methanol upon crystallization. ^1H NMR (400 MHz, $CDCl_3$, 25 ^oC, TMS): δ = 8.84 (s, 1H), 7.63 (t, J = 7.6 Hz, 1H), 7.57 (t, J = 8.4 Hz, 1H), 7.36-7.29 (m, 9H), 7.22 (t, J = 7.6 Hz, 3H), 6.91 (d, J = 7.6 Hz, 2H), 2.53-2.40 (m, 4H), 1.35 (d, J = 6.8 Hz, 6H), 1.00 (d, J = 6.8 Hz, 6H), 0.85-0.83 (m, 12 H) ppm; ^{13}C NMR (100 MHz, $CDCl_3$, 25 ^oC, TMS): δ = 145.1, 144.8, 144.4, 137.0, 132.9, 132.8, 132.4, 130.8, 130.0, 129.6, 129.4, 129.2, 128.6, 126.1, 125.4, 124.3, 123.5, 120.6, 36.3, 29.4, 29.1, 25.6, 23.7, 23.3, 22.5, 21.9, 20.0 ppm. HRMS m/z (ESI) calc. for $C_{39}H_{49}ClN_2$ $[M+H]^+$ 545.3879, found 545.3862.

Expulsion of carbon dioxide from compound 3 under heating

In the open atmosphere, solid compound **3** (10.0 mg, 0.014 mmol) was heated at 150 ^oC for 12 h in a glass vial. During this process, we observed a sharp color change from colorless to chocolate color. After 12 h, the final compound was washed with hexane for three times. ^1H NMR (500 MHz, $CDCl_3$, 25 ^oC, TMS): δ = 8.36 (s, 1H), 7.98-7.92 (m, 2H), 7.64-7.57 (m, 2H), 7.43-7.40 (m, 2H), 7.37-7.29 (m, 7H), 7.24-7.21 (m, 7H), 6.93 (d, J = 7.5 Hz, 2H), 3.14 (sept, J = 6.5 Hz, 1H), 2.52-2.39 (m, 4H), 1.88-1.83 (m, 7H), 1.64-1.56 (m, 6H), 1.29 (d, J = 7.0 Hz, 6H), 1.22 (d, J = 7.0 Hz, 3H), 1.00 (d, J = 6.5 Hz, 6H), 0.85-0.84 (m, 12H) ppm; ^{13}C NMR (125 MHz, $CDCl_3$, 25 ^oC, TMS): δ = 144.8, 144.4, 132.8, 132.5, 129.6, 129.5, 129.2, 128.5, 128.0,

127.8, 127.5, 127.1, 126.1, 125.4, 71.6, 36.3, 29.5, 29.4, 29.2, 25.4, 23.8, 23.5, 23.3, 22.5, 20.0 ppm. ^{11}B NMR (160 MHz, $CDCl_3$, 25 ^{o}C, TMS): δ = 20.9 ppm

Synthesis of {1,3-bis(2,6-diisopropylphenyl)-2,4-diphenylimidazolidine bicarbonate boric acid} (5)

The compound **2** (20.0 mg, 0.029 mmol) was exposed as a fine powder in a petridish for 3 days in an open atmosphere at room temperature. From this air-exposed solid, colorless crystals of compound **5** {1,3-bis(2,6-diisopropylphenyl)-2,4-diphenylimidazolidine bicarbonate boric acid}(9.5 mg, 0.0145mmol, 50%) were grown using a benzene/*n*-hexane solvent mixture.^{1}H NMR (500 MHz, $CDCl_3$, 25 ^{o}C, TMS): δ = 8.77 (s, 1H), 8.58 (s, 0.5H), 7.41-7.29 (m, 11H), 7.24-7.20 (m, 2H), 6.94 (d, J = 7.5 Hz, 2H), 2.55-2.42 (m, 4H), 1.34 (d, J = 6.5 Hz, 6H), 1.01 (d, J = 7.0 Hz, 6H), 0.87-0.85 (m, 12H) ppm; ^{13}C NMR (125 MHz, $CDCl_3$, 25 ^{o}C, TMS): δ = 169.0, 144.9, 144.5, 137.1, 132.7, 132.4, 132.2, 130.9, 129.7, 129.5, 129.2, 129.1, 128.6, 127.8, 127.1, 126.1, 125.4, 124.8, 124.6, 123.7, 120.8, 29.5, 29.2, 25.4, 23.8, 23.4, 22.6 ppm; ^{11}B NMR (160 MHz, $CDCl_3$, 25 ^{o}C, TMS): δ = 21.0 ppm. Elemental analysis: Calcd. for$C_{40}H_{53}BN_2O_6$: C 71.85, H 7.99, N 4.19; found: C 73.72, H 8.19, N 4.24.

Details of TGA study

In order to have a clear picture on expulsion of CO_2 molecule from the compound **3**, we have performed thermogravimetric experiment using NETSZCH TGA-DTA system upon heating from 27 to 550 °C ramp at 4°C/min. According to the thermal gravimetric curve (black) in Fig. 3d, it is seen that first significant weight loss starts from 95.5 °C continue up to 160.2°C. This corresponds to the weight loss of 7.22% (M.W. 651.4 to 603.9). This is

slightly high to the mass loss of CO_2 per mole or per formula unit (theoretical value 6.76% for M.W. 651.4 to 607.4). Differential Thermal Analysis (DTA) curve (blue) in Fig. 3d also shows the complete exothermic loss of CO_2 molecule in this particular region. From the DTA curve, it is convincing that complete weight loss of CO_2 lasts up to around 160 °C assuming that decomposing volatiles obey first order kinetics. So, it is concluded from the gravimetric analysis that heating the compound at 150 °C can result in the release of carbon dioxide from **3**.

Computational details

All optimization were performed using M062X[3-4]/6-31+G(d,p)[5] level of theory as M062X is reported to be highly efficient for main group elements. Gaussian 09[6]suit of programs is employed for all calculation. As reported earlier, the ground state of *a*NHCcarbene (**1**) is singlet and the first triplet state is 49.8 kcal/mol higher in energy.[7] ΔE_{ST} were calculated using B3LYP[8-9]DFT functional along with same basis set. All the ground state minima are characterized by absence of imaginary frequency while the transition states are confirmed by presence of one unique imaginary frequency that connects pertinent reactant and products.For the incorporation of solvent effect, a polarizable continuum model based on SMD has been used. All the free energy energies are obtained using SMD/M062X/6-31+G(d,p)//M062X/6-31+G(d,p) level of theory. Few structures are optimized utilizing two layer ONIOM[10-12]method where M062X/6-31+G(d,p) is used for high layer and AM1[13] is used for low layer. Layer specification for the ONIOM method is shown below.

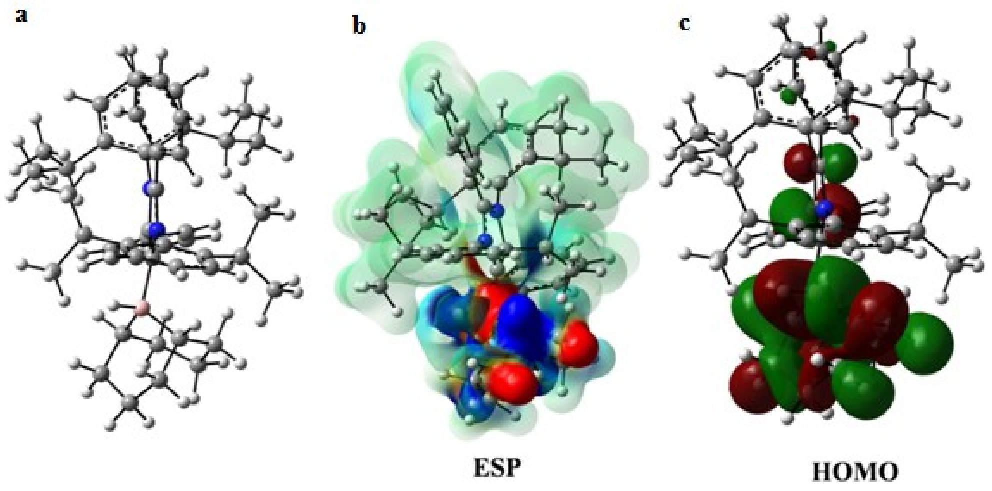

Fig. S1 Theoretical investigation of compound **2**.a)Optimized geometry of **2**.b) Electrostatic potential surface of **2**. c)Highest Occupied Molecular Orbital (HOMO) (iso=0.04) of **2**.

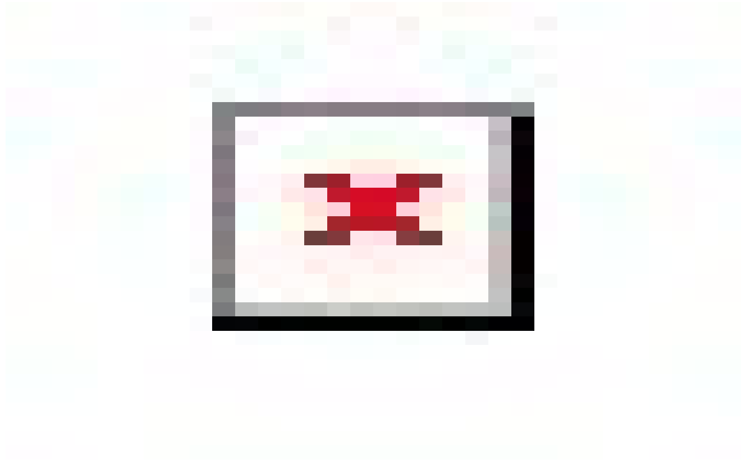

Fig. S2 A tube model is used to describe the high LEVEL layer, which has been treated quantum mechanically and the LOW LEVEL layer (shown with a wireframe model) has been treated with a semi-empirical method.

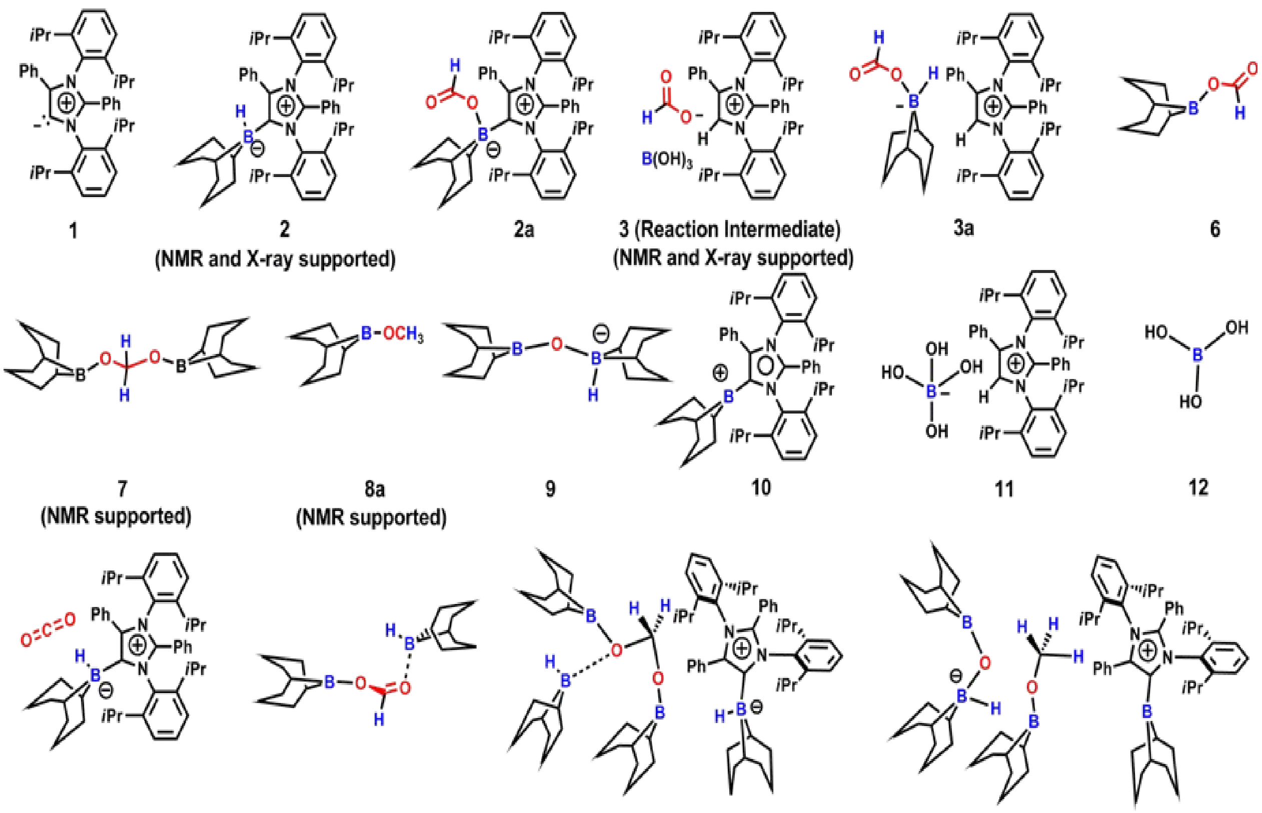

Fig. S3 Structural drawings of compounds involving the proposed mechanism depicted in Figures 6 and 7 of the main manuscript.

Fate of intermediate 10 in presence of moisture

In step 6 (Fig.6, manuscript), during hydride transfer from **2** to **7**, the intermediate **10** is released. Since this is an open atmosphere reaction, water reacts with **10** to form a weak reactant complex **13** that undergoes O-H bond cleavage of water *via***TS6**(Figure S3) and generates 9BBN-OH (**14**) along with the release of the carbene salt (**15**). The activation energy for the decomposition is computed to be 25.6 kcal/mol and it goes through a

transition state namely **TS6**. The overall exergonicity of the process is 28.7 kcal/mol (ΔG =-28.7 kcal/mol). All the free energies in the bracket are given in kcal/mol. Relevant bond distances are shown in Å.

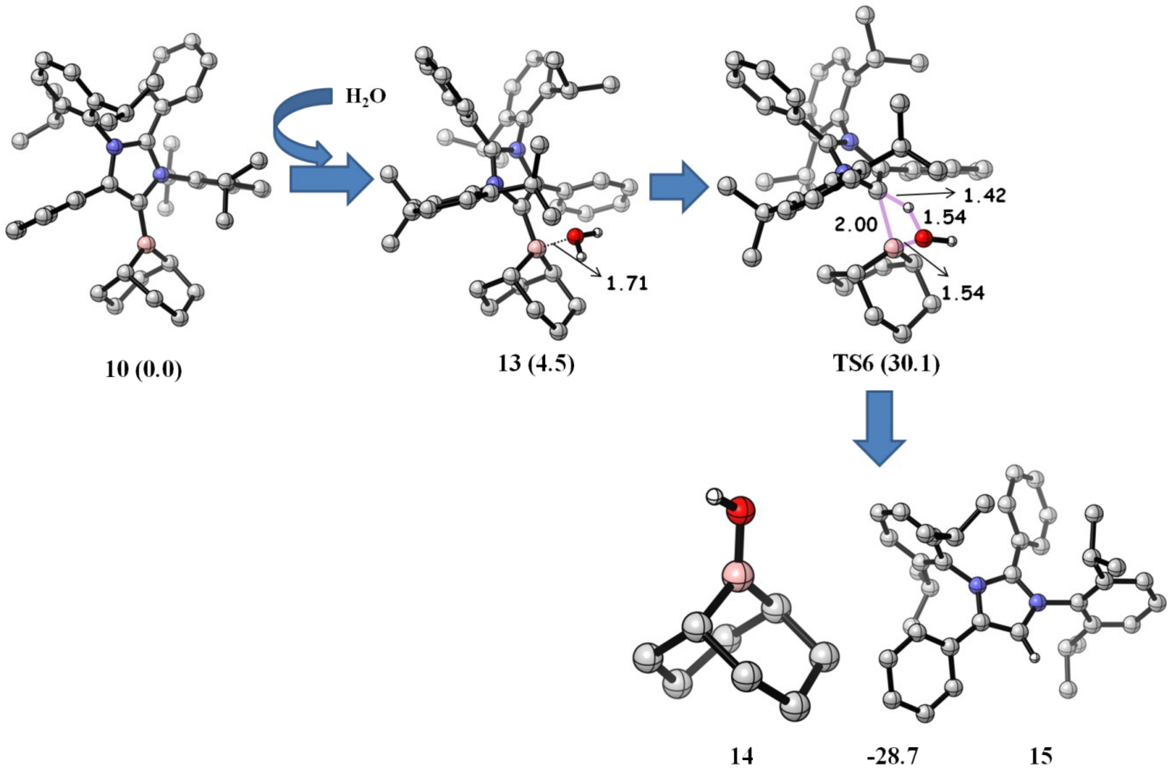

Fig. S4 Optimized structures for conversion of compound **10** to **14** and **15** *via* TS6.

Details for preparation of video clipping of the carbon dioxide reduction from air with compound 3

With the help of a glovebox,9-BBN(0.035 mmol, 10 equiv) was added in a 5 mL glass vial containing compound **3** (5.0 mg, 0.007 mmol) and the vial was taken out of the glovebox. To the vial, quickly 2 mL C_6D_6 was added in open air and the cap of the vial was closed and kept. Immediately, we observed a gas evolution, which was confirmed as hydrogen by ^1H

NMR spectroscopy (δ = 4.46 ppm).[14] Although the total time of the reaction to consume the substrates is about 6 h, we have presented the edited video file capturing visual gas evaluation during the reaction initial thirty seconds when gas evolution rate was very high. In this video, especially we have captured the gas evolution first few seconds after addition of 9-BBN, when the rate of gas evolution was very high and the rate becomes slow with time (after 30 min. the rate of evolution becomes very slow).

X-ray crystal structure measurements

Suitable single crystals of **2**, **3** and **5** were selected and mounted under nitrogen atmosphere using the X-TEMP2 and intensity data were collected on a Super Nova, Dual, Cu at zero, Eos diffractometer. The crystal was kept at 100.00(10) K during data collection. Using Olex2,[15] the structure was solved with the Superflip[16]structure solution program using Charge Flipping and refined with the ShelXL[17] refinement package using Least Squares minimization. All non-hydrogen atoms were refined with anisotropic displacement parameters. The hydrogen atoms were refined isotropically on calculated positions using a riding model. Disordered moieties were refined using bond lengths restraints and isotropic displacement parameters restraints. Crystallographic data (excluding structure factors) for the structures have been deposited with the Cambridge Crystallographic Data Centre. Single-crystal X-ray structural studies of compound **4** were performed on an Oxford Diffraction XCALIBUR-EOS CCD equipped diffractometer with an Oxford Instruments low temperature attachment. Crystal data were collected at 293(2) K using graphite-monochromated Mo Kαradiation (λα = 0.71073 Å). The strategy for the data collection was evaluated by using the CrysAlisPro CCD software. The data were collected by the standard

'ψ–ω' scan method and were scaled and reduced using CrysAlisPro RED software. The structures were solved by direct methods (SHELXS) and refined by full-matrix least-squares calculations on F^2 (SHELXL).[18]Copies of the data can be obtained free of charge from The Cambridge Crystallographic Data Centre *via* www.ccdc.cam.ac.uk/data_request/cif. CCDC 1583077, 1583079, 1583078 and 1583080contains the supplementary crystallographic data of compounds **2**, **3**, **4** and **5** respectively for this paper.

Table S1. Crystallographic and data collection parameters for compounds **2**-**5**.

	$C_{47}H_{59}BN_2$, **2**	$C_{43}H_{52}B\ N_2O_5$,**3**	$C_{39}H_{47}N_2ClO$,**4**	$C_{40}H_{48}BN_2O$, **5**
Formula weight	662.77	687.68	595.24	633.75
Temperature	100.00 K	293.00 K	295	100 K
Crystal system	Monoclinic	monoclinic	orthorhombic	orthorhombic
Space group	P21/n	P21/n	Pna21	P21212
a (Å)	11.2461(3)	12.6081(5)	18.4291(7)	19.3779(19)
b (Å)	23.0691(6)	14.3388(6)	13.1243(4)	14.5134(16)
c (Å)	15.3687(3)	22.0714(7)	14.5076(6)	13.085(2)
α (°)	90.000	90.00	90.00	90.00
β (°)	98.7643(19)	98.028(4)	90.00	90.00
γ (°)	90.000	90.00	90.00	90.00
V(Å^3)	3940.66(17)	3951.1(3)	3508.9(2)	3680.0(8)

Z	4	4	4	4
D_{calc}(g/cm^3)	1.117	1.156	1.127	1.144
F (000)	1440	1476	1280	1360
μ (mm^{-1})	0.063	0.074	0.140	0.074
θ Range (°)	2.0-28.1	1.7-25.0	4.4-29.2	1.8-25.0
Goodness-of-fit	1.07	1.07	1.01	1.06
R1, wR2 [I>2σ(I)]	0.0494, 0.1251	0.0527, 0.1220	0.0463, 0.1208	0.0845, 0.2180
Largest difference in peak and hole (e/ Å^3)	0.28 and -0.29	0.22 and -0.18	0.26 and -0.21	0.29 and -0.48

Table S2. Selected bond distances (Å) and angles (°) for compounds **2**-**5**.

Compound **2**			
	Bond distances (Å)		Bond angles (°)
C5 -B1	1.636(2)	C5 -B1 -C42	111.13(13)
B1 -H1	0.9800	C5 -B1 -C46	116.74(14)
C46 -B1	1.631(2)	C42 -B1 -C46	104.07(13)
C42 -B1	1.639(2)	N1 -C5 -B1	121.94(13)
N1 -C5	1.415(2)	C4 -C5 -B1	134.35(14)
C4 -C5	1.368(2)	N1 -C5 -C4	103.71(13)
		C5 -B1 -H1	108.00
		C42 -B1 -H1	108.00
		C46 -B1 -H1	108.00
Compound **3**			
O1 -C1	1.242(3)	O1 -C1 -O2	127.6(2)
O2 -C1	1.260(3)	O3 -B1 -O4	118.4(2)
O3 -B1	1.363(4)	O3 -B1 -O5	117.9(3)
O4 -B1	1.375(4)	O4 -B1 -O5	123.7(3)
O5 -B1	1.358(4)	N1 -C5 -C4	108.8(2)
C4 -C5	1.355(3)		

N1 -C5	1.383(3)		
Compound **4**			
N1 -C5	1.376(3)	N1 -C5 -C4	107.94(16)
C4 -C5	1.356(3)	N3 -C4 -C5	105.77(16)
N3 -C2	1.348(2)	N1 -C2 -N3	106.88(16)
N1 -C2	1.344(2)	C2 -N3 -C4	109.66(14)
N3 -C4	1.402(2)	N1 -C5 -H5	126.00
C5 -H5	0.9300	C4 -C5 -H5	126.00
Compound **5**			
O1 -C42	1.323(11)	O1 -C42 -O3	111.4(7)
O2 -C42	1.340(9)	O2 -C42 -O3	124.3(7)
O3 -C42	1.338(10)	O1 -C42 -O2	124.3(8)
O4 -B1	1.356(7)	O4 -B1 -O5	121.9(4)
O5 -B1	1.329(12)	O4 -B1 -O6	116.3(8)
O6 -B1	1.356(7)	O6 -B1 -O5	121.9(4)
N1 -C5	1.371(6)	N1 -C5 -C4	109.1(4)
C4 -C5	1.326(7)		

Table S3. Final coordinates and equivalent isotropic displacement parameters of the non-hydrogen atoms for compound **2**.

**Atom**	***x***	***y***	***Z***	**U(eq)**
N3	8247.0(11)	1221.2(5)	2478.5(8)	13.8(3)
N1	10015.4(11)	1486.7(6)	2261.5(8)	13.8(3)
C31	11345.3(15)	1434.6(7)	1155.2(11)	19.6(3)
C30	11228.8(14)	1445.9(7)	2053.8(11)	16.2(3)
C17	6623.6(14)	528.5(7)	2047.8(11)	16.9(3)
C6	7005.2(14)	2106.4(7)	2037.8(10)	14.9(3)
C12	7242.9(13)	887.9(7)	2691.4(10)	14.8(3)
C13	6976.1(14)	934.8(7)	3551.4(10)	16.2(3)

C2	9368.5(14)	1021.2(7)	2456.4(10)	14.6(3)
C5	9310.5(14)	1997.1(7)	2149.2(10)	14.8(3)
C15	5398.6(15)	230.9(7)	3130.4(12)	22.3(4)
C24	9759.9(14)	408.9(7)	2571.7(10)	16.6(3)
C29	10684.8(15)	187.0(7)	2147.9(11)	19.8(3)
C35	12199.5(15)	1393.7(7)	2736.4(11)	19.2(3)
C11	6243.4(15)	2240.5(7)	2644.9(11)	18.7(3)
C21	7701.1(15)	1320.3(7)	4243.6(10)	18.9(3)
C4	8188.1(14)	1813.2(6)	2263.1(10)	14.2(3)
C18	6948.6(15)	477.8(7)	1124.5(11)	20.1(4)
C49	11677.0(14)	3318.4(7)	2382.0(11)	20.0(3)
C10	5149.5(15)	2511.2(7)	2378.6(11)	20.9(4)
C26	9542.9(16)	-552.0(7)	3171.7(12)	24.2(4)
C32	12502.2(17)	1363.4(8)	956.3(12)	25.8(4)
C43	10558.0(16)	2904.4(8)	3584.8(11)	23.1(4)
C19	5942.8(16)	706.1(8)	427.0(11)	25.8(4)
C16	5685.0(15)	200.8(7)	2290.7(12)	21.1(4)
C42	11008.3(14)	2798.4(7)	2704.1(10)	17.4(3)
C28	11027.9(16)	-390.1(8)	2246.5(12)	24.5(4)
C46	8989.8(15)	3183.3(7)	1888.2(11)	18.5(3)
C45	8583.1(15)	3327.4(8)	2777.9(12)	23.9(4)
C48	10890.2(15)	3843.0(7)	2063.8(12)	20.8(4)
C7	6640.1(15)	2256.6(7)	1156.8(11)	19.0(3)

C44	9593.5(16)	3372.9(8)	3567.0(11)	25.5(4)
C14	6036.6(15)	593.7(7)	3755.5(11)	20.5(4)
C9	4797.6(15)	2655.4(7)	1502.0(11)	22.5(4)
C25	9202.9(15)	25.7(7)	3093.2(11)	20.9(4)
C47	9660.6(15)	3690.5(7)	1524.7(12)	21.8(4)
C8	5543.8(16)	2530.9(8)	893.5(11)	24.2(4)
C36	12079.3(16)	1408.9(8)	3708.4(11)	24.0(4)
C33	13481.1(17)	1305.1(8)	1613.9(14)	29.9(4)
C23	6922.8(17)	1574.4(8)	4884.9(12)	27.3(4)
C27	10460.6(17)	-762.2(8)	2753.8(12)	27.0(4)
C34	13332.0(16)	1321.3(8)	2487.2(13)	26.6(4)
B1	9884.7(16)	2623.9(8)	1939.8(12)	16.2(4)
C41	9844.2(17)	861.9(8)	97.8(12)	27.9(4)
C22	8779.8(17)	1002.4(9)	4762.4(12)	29.1(4)
C20	7269.1(17)	-148.8(8)	920.8(12)	26.6(4)
C37	12952.9(18)	1845.7(9)	4220.8(13)	33.3(5)
C38	12293.6(18)	810.7(9)	4138.0(13)	32.1(4)
C40	10514(2)	1831.3(9)	-358.5(13)	41.4(5)
C39	10269.7(17)	1464.4(8)	423.5(11)	23.6(4)

Table S4. Hydrogen atom positions and isotropic displacement parameters for compound **2**.

Atom	*x*	*y*	*z*	*U(eq)*
H15	4773	6	3279	27
H29	11072	430	1797	24
H11	6473	2147	3235	22
H21	8015	1645	3935	23
H18	7663	717	1102	24
H49A	12291	3443	2858	24
H49B	12084	3190	1903	24
H10	4649	2597	2790	25
H26	9149	-801	3509	29
H32	12620	1355	370	31
H43A	10237	2544	3775	28
H43B	11240	3011	4021	28
H19A	5220	490	453	39
H19B	6173	663	-146	39
H19C	5804	1108	536	39
H16	5245	-42	1879	25
H42	11567	2470	2782	21
H28	11649	-528	1967	29
H46	8271	3097	1462	22

H45A	8151	3693	2718	29
H45B	8023	3031	2905	29
H48A	11328	4086	1706	25
H48B	10756	4068	2573	25
H7	7136	2172	742	23
H44A	9972	3750	3556	31
H44B	9244	3348	4105	31
H14	5833	609	4319	25
H9	4061	2835	1325	27
H25	8597	161	3390	25
H47A	9780	3596	929	26
H47B	9152	4032	1492	26
H8	5313	2631	306	29
H36	11257	1530	3759	29
H33	14246	1255	1466	36
H23A	6185	1719	4561	41
H23B	7348	1886	5210	41
H23C	6748	1278	5285	41
H27	10692	-1149	2814	32
H34	14002	1283	2920	32
H1	10192	2593	1378	19
H41A	10474	671	-150	42
H41B	9146	898	-344	42

H41C	9646	638	582	42
H22A	8503	665	5037	44
H22B	9188	1256	5206	44
H22C	9323	888	4369	44
H20A	7931	-277	1347	40
H20B	7491	-167	343	40
H20C	6586	-394	945	40
H37A	13766	1718	4221	50
H37B	12795	1872	4816	50
H37C	12843	2219	3945	50
H38A	11727	539	3839	48
H38B	12191	834	4746	48
H38C	13097	684	4098	48
H40A	10829	2201	-151	62
H40B	9778	1886	-757	62
H40C	11088	1637	-658	62
H39	9608	1648	668	28

Table S5. Final coordinates and equivalent isotropic displacement parameters of the non-hydrogen atoms for compound **3**.

**Atom**	***x***	***y***	***Z***	**U(eq)**
O2	1958.6(16)	8892.6(14)	4592.5(8)	31.9(5)

O5	5638.7(17)	2307.8(14)	4875.6(10)	39.4(6)
N1	5285.1(16)	5546.8(14)	8017.8(9)	16.7(5)
O4	6036.7(16)	678.2(14)	5049.5(9)	33.6(5)
O1	2226.6(17)	7407.2(14)	4896.1(9)	40.3(6)
O3	4261.3(15)	1217.9(14)	4847.6(10)	36.8(5)
N3	6162.7(16)	5530.3(14)	7237.9(9)	15.3(5)
C36	8111(2)	5519.0(18)	7788.8(11)	18.6(6)
C35	4929(2)	3310(2)	6250.8(13)	32.3(7)
C4	6932(2)	5525.7(17)	7762.7(11)	16.7(6)
C2	5175(2)	5528.7(17)	7396.1(11)	16.1(6)
C12	4775(2)	4007.5(19)	8802.7(12)	25.7(7)
C30	6771(2)	7284.6(18)	6729.5(12)	25.3(7)
C29	6558(2)	6401.6(19)	6341.8(12)	22.0(7)
C25	6167(2)	4705.6(18)	6265.7(11)	20.0(6)
C40	9766(2)	5561(2)	7350.0(13)	31.1(7)
C33	5955(2)	3784.4(18)	6565.2(12)	23.9(6)
C24	6331.3(19)	5544.0(18)	6593.6(11)	16.8(6)
C5	6366(2)	5546.5(17)	8241.1(11)	18.9(6)
C37	8723(2)	5514.5(19)	8369.6(12)	25.2(7)
C19	3936(2)	5958.9(18)	6434.2(11)	19.2(6)
C15	4242(2)	7349.9(19)	7989.4(12)	26.3(7)
C18	4130(2)	5479.8(17)	6995.4(11)	17.7(6)
C7	3944(2)	6551.4(19)	8383.9(11)	22.9(6)

C23	3323(2)	4946.8(18)	7185.1(11)	20.0(6)
C28	6583(2)	6410(2)	5712.3(12)	26.8(7)
C6	4447(2)	5677.9(19)	8397.9(11)	20.3(6)
C20	2937(2)	5903.5(18)	6086.3(12)	22.7(6)
C32	5802(2)	7931.1(19)	6675.2(13)	30.7(7)
C14	5487(2)	3917(2)	9426.3(12)	29.7(7)
C26	6221(2)	4763(2)	5638.6(12)	27.2(7)
C41	8651(2)	5527(2)	7279.4(12)	27.9(7)
C11	4194(2)	4938.1(19)	8770.1(11)	22.4(6)
C27	6413(2)	5603(2)	5369.0(12)	29.4(7)
C38	9828(2)	5534(2)	8433.8(13)	29.7(7)
C22	2322(2)	4904.0(19)	6835.7(12)	24.7(7)
C1	1667(2)	8066(2)	4669.4(12)	30.3(7)
C34	6925(2)	3139(2)	6581.0(13)	35.2(8)
C21	2129(2)	5380.8(19)	6283.1(12)	25.7(7)
C8	3148(2)	6668(2)	8757.7(12)	30.1(7)
C17	4768(2)	8154(2)	8380.7(13)	32.5(7)
C31	7746(2)	7824(2)	6571.0(16)	43.3(9)
C9	2872(2)	5954(2)	9125.1(12)	32.8(8)
C16	3256(2)	7682(2)	7549.1(13)	33.8(8)
C10	3388(2)	5102(2)	9127.9(12)	28.5(7)
C13	4000(2)	3176(2)	8695.0(13)	37.9(8)
C39	10352(2)	5567(2)	7925.1(13)	30.3(7)

C43	792(2)	5431(2)	4721.6(13)	36.8(8)
C42	97(2)	5958(2)	5010.6(13)	37.0(8)
B1	5331(3)	1407(2)	4925.9(14)	30.5(8)
C44	-693(2)	5526(2)	5286.3(13)	37.4(8)

Table S6. Hydrogen atom positions and isotropic displacement parameters for compound **3**.

Atom	x	y	z	U(eq)
H5A	6292	2344	4955	59
H3	4170	652	4859	55
H35A	4332	3726	6252	48
H35B	4802	2750	6468	48
H35C	5012	3158	5836	48
H12	5241	3999	8482	31
H30	6930	7091	7158	30
H40	10117	5580	7006	37
H33	5855	3915	6989	29
H5	6657	5559	8652	23
H37	8381	5498	8716	30
H19	4476	6310	6298	23
H15	4769	7113	7740	32
H23	3454	4615	7550	24

H28	6717	6966	5520	32
H20	2807	6221	5716	27
H32A	5628	8135	6259	46
H32B	5965	8463	6936	46
H32C	5202	7603	6797	46
H14A	6009	4409	9470	45
H14B	5847	3325	9449	45
H14C	5051	3960	9748	45
H26	6127	4228	5400	33
H41	8264	5508	6889	33
H27	6427	5627	4949	35
H38	10222	5525	8823	36
H22	1779	4556	6971	30
H1	948	7934	4541	36
H34A	6791	2568	6784	53
H34B	7546	3439	6798	53
H34C	7048	3005	6170	53
H21	1458	5348	6047	31
H8	2796	7237	8759	36
H17A	4293	8364	8656	49
H17B	4915	8659	8119	49
H17C	5426	7941	8611	49
H31A	8354	7416	6602	65

H31B	7898	8335	6851	65
H31C	7595	8060	6161	65
H9	2339	6045	9371	39
H16A	2958	7167	7305	51
H16B	3468	8162	7287	51
H16C	2728	7925	7781	51
H10	3191	4626	9376	34
H13A	4403	2607	8700	57
H13B	3562	3244	8305	57
H13C	3553	3158	9013	57
H39	11096	5594	7971	36
H43	1322	5719	4534	44
H42	159	6605	5020	44
H44	-1161	5885	5479	45
H4	6701(15)	840(20)	5170(16)	58(11)

Table S7. Final coordinates and equivalent isotropic displacement parameters of the non-hydrogen atoms for compound **4**.

Atom	x	y	z	U(eq)
N3	-4539.0(8)	-2222.1(10)	-4022.4(11)	37.0(3)
N1	-4355.9(8)	-3714.8(11)	-3436.6(12)	40.9(4)
C29	-4264.1(13)	-415.4(15)	-3816.7(14)	50.6(5)

C4	-4111.2(10)	-2763.1(13)	-4654.3(13)	37.9(4)
C5	-3998.4(10)	-3691.5(14)	-4269.3(14)	42.4(4)
C18	-5138.3(11)	-2568.9(15)	-2483.9(13)	44.2(5)
C24	-4767.7(11)	-1156.7(13)	-4070.2(13)	40.9(4)
C2	-4686.2(10)	-2816.8(14)	-3288.5(13)	38.5(4)
C25	-5482.9(12)	-951.4(15)	-4329.6(13)	46.5(5)
C6	-4341.9(12)	-4557.7(15)	-2780.3(15)	49.6(5)
C15	-5308.3(14)	-5423.9(19)	-3768.8(19)	65.4(7)
C36	-3847.3(10)	-2385.0(14)	-5548.8(13)	40.0(4)
C38	-3892.5(14)	-1273.8(19)	-6867.8(16)	61.5(6)
C7	-4831.8(14)	-5360.4(16)	-2922.2(18)	59.2(6)
C22	-6083.0(14)	-3041(2)	-1422.5(18)	66.6(7)
C23	-5670.2(12)	-3247.0(17)	-2190.1(16)	53.0(5)
C19	-5044.4(15)	-1667.8(18)	-1995.6(15)	59.5(6)
C30	-3478.5(15)	-651(2)	-3590.3(19)	68.3(7)
C37	-4168.7(13)	-1586.6(16)	-6022.3(15)	52.8(5)
C33	-5999.3(12)	-1749.1(17)	-4683.9(18)	61.2(6)
C28	-4519.5(17)	590.3(16)	-3805.1(17)	65.6(7)
C41	-3262.5(12)	-2881.9(19)	-5948.9(16)	56.5(6)
C27	-5225.9(17)	806.7(17)	-4027(2)	72.0(8)
C40	-2998.8(14)	-2568(2)	-6797.5(17)	67.6(7)
C8	-4849(2)	-6108(2)	-2253(2)	83.6(9)
C26	-5704.9(14)	69.5(16)	-4287.8(17)	60.4(6)

C39	-3309.0(15)	-1763(2)	-7246.7(17)	65.8(6)
C11	-3855.3(14)	-4495(2)	-2057.8(18)	65.1(7)
C9	-4403(2)	-6057(2)	-1513(3)	99.2(11)
C21	-5974.1(16)	-2155(2)	-941.8(17)	73.7(8)
C34	-6259.4(16)	-1485(3)	-5655(2)	86.8(9)
C17	-6069.0(17)	-5846(3)	-3569(3)	99.0(11)
C16	-4934.2(17)	-6070(2)	-4508(2)	86.7(9)
C10	-3901.6(19)	-5292(3)	-1407(2)	92.6(10)
C20	-5465.8(18)	-1467(2)	-1229.2(17)	74.8(8)
C31	-2973.7(17)	-256(3)	-4340(2)	96(1)
C12	-3284.2(17)	-3686(3)	-1975(2)	88.3(10)
C32	-3239(2)	-231(3)	-2652(3)	114.5(14)
C35	-6660.4(16)	-1890(3)	-4054(2)	86.9(9)
C14	-2529(2)	-4218(4)	-2077(4)	136.7(16)
C13	-3337(2)	-3074(3)	-1089(3)	114.2(12)
Cl1	-2728.1(3)	-5549.7(5)	-4789.0(7)	83.4(2)
O1	-3293(2)	-7338(2)	-3543(3)	157.8(15)

Table S8. Hydrogen atom positions and isotropic displacement parameters for compound **4**.

**Atom**	**x**	**y**	**z**	**U(eq)**
H5	-3727	-4219	-4523	51

H15	-5365	-4733	-4014	78
H38	-4105	-730	-7177	74
H22	-6435	-3501	-1230	80
H23	-5748	-3847	-2517	64
H19	-4697	-1199	-2185	71
H30	-3427	-1394	-3569	82
H37	-4571	-1259	-5773	63
H33	-5739	-2399	-4714	73
H28	-4206	1117	-3645	79
H41	-3046	-3428	-5646	68
H27	-5384	1479	-3998	86
H40	-2608	-2908	-7063	81
H8	-5170	-6651	-2311	100
H26	-6179	244	-4439	72
H39	-3123	-1547	-7810	79
H9	-4438	-6557	-1061	119
H21	-6248	-2021	-417	88
H34A	-6561	-889	-5631	130
H34B	-6533	-2045	-5900	130
H34C	-5848	-1355	-6043	130
H17A	-6033	-6548	-3391	149
H17B	-6363	-5790	-4112	149
H17C	-6287	-5462	-3077	149

H16A	-4469	-5781	-4649	130
H16B	-5228	-6082	-5054	130
H16C	-4871	-6753	-4283	130
H10	-3590	-5300	-903	111
H20	-5403	-860	-907	90
H31A	-3121	-527	-4925	144
H31B	-2485	-465	-4209	144
H31C	-2997	475	-4358	144
H12	-3343	-3214	-2493	106
H32A	-3227	500	-2676	172
H32B	-2764	-484	-2507	172
H32C	-3576	-445	-2186	172
H35A	-6500	-2042	-3440	130
H35B	-6953	-2442	-4280	130
H35C	-6942	-1275	-4048	130
H14A	-2434	-4626	-1541	205
H14B	-2157	-3710	-2139	205
H14C	-2531	-4646	-2614	205
H13A	-3823	-2826	-1015	171
H13B	-3007	-2509	-1116	171
H13C	-3213	-3502	-575	171
H1A	-3147	-6871	-3182	237
H1B	-3723	-7206	-3719	237

Table S9. Final coordinates and equivalent isotropic displacement parameters of the non-hydrogen atoms for compound **5**.

Atom	x	y	z	U(eq)
O4	123(2)	4224(3)	9763(3)	45.4(11)
N1	1932(2)	3004(3)	8485(3)	26.2(10)
N3	2139(2)	3661(3)	7033(3)	27.5(10)
O2	5128(3)	2279(3)	9003(3)	58.0(13)
O3	5639(2)	1371(4)	7704(4)	54.8(12)
O5	0	5000	11326(4)	56.5(19)
C24	2748(2)	2142(4)	7346(4)	28.2(12)
C5	1584(2)	3826(3)	8476(4)	27.4(11)
C6	1473(3)	5168(4)	7242(4)	29.5(12)
C12	2317(3)	3738(4)	5941(4)	27.3(11)
C2	2275(2)	2913(3)	7599(4)	24.7(11)
C13	1797(3)	3543(4)	5235(4)	29.9(12)
C11	1829(3)	5719(4)	6574(4)	36.2(13)
C16	3141(3)	3998(4)	4624(5)	38.0(14)
C30	1884(3)	2289(4)	9273(4)	32.5(13)
C4	1711(3)	4258(3)	7603(4)	29.3(12)
C31	2271(3)	2407(4)	10169(4)	30.5(12)
C35	1479(3)	1510(4)	9059(4)	29.4(12)
C18	1058(3)	3290(4)	5530(4)	32.3(13)

C17	3000(3)	3963(4)	5667(4)	29.0(12)
C25	2692(3)	1643(4)	6431(4)	31.2(12)
C32	2235(3)	1681(4)	10878(5)	41.2(14)
C7	849(3)	5507(4)	7673(5)	36.0(14)
C29	3264(3)	1946(4)	8050(4)	34.0(13)
C15	2645(3)	3800(4)	3902(5)	36.8(13)
C27	3655(3)	692(4)	6951(5)	42.0(15)
C36	1023(3)	1428(4)	8136(4)	33.5(13)
C33	1857(3)	918(4)	10695(5)	39.8(14)
C21	3573(3)	4150(4)	6424(5)	38.5(14)
C39	2684(3)	3280(4)	10390(5)	35.5(13)
C41	2244(3)	3949(4)	11024(5)	41.4(15)
C28	3707(3)	1216(4)	7844(5)	41.9(15)
C9	971(3)	6922(4)	6713(5)	40.5(14)
C14	1971(3)	3576(4)	4208(5)	34.0(13)
C38	1149(3)	544(4)	7544(5)	41.1(15)
C26	3142(3)	925(4)	6240(4)	36.1(13)
C34	1471(3)	826(4)	9819(5)	41.1(14)
C8	610(3)	6357(4)	7412(5)	39.7(14)
C23	4175(3)	3458(4)	6273(5)	38.7(14)
C40	3371(3)	3051(5)	10941(5)	43.5(15)
B1	0	5000	10310(8)	46(3)
C20	540(3)	3982(4)	5088(5)	42.8(15)

C42	5499(4)	2181(6)	8146(6)	56(2)
C37	246(3)	1501(5)	8468(5)	46.7(16)
C19	856(3)	2316(4)	5180(5)	42.7(15)
C10	1585(3)	6586(4)	6305(5)	41.4(15)
O1	5762(4)	2874(5)	7618(6)	100(2)
C22	3854(3)	5133(4)	6320(6)	54.0(18)

Table S10. Hydrogen atom positions and isotropic displacement parameters for compound **5**.

**Atom**	**x**	**y**	**z**	**U(eq)**
H4	130(40)	3779(8)	10149(8)	68.1(16)
H5a	50(100)	4472(18)	11535(4)	85(3)
H5	1304(2)	4046(3)	8999(4)	32.9(14)
H11	2242(3)	5509(4)	6296(4)	43.4(16)
H16	3582(3)	4160(4)	4409(5)	45.6(17)
H18	1023(3)	3312(4)	6277(4)	38.8(15)
H25	2353(3)	1795(4)	5956(4)	37.5(15)
H32	2477(3)	1728(4)	11489(5)	49.4(17)
H7	603(3)	5147(4)	8135(5)	43.2(16)
H29	3312(3)	2294(4)	8643(4)	40.8(16)
H15	2757(3)	3815(4)	3211(5)	44.2(16)
H27	3954(3)	203(4)	6831(5)	50.4(18)

H36	1125(3)	1948(4)	7682(4)	40.2(15)
H33	1857(3)	444(4)	11173(5)	47.8(17)
H21	3389(3)	4073(4)	7116(5)	46.2(17)
H39	2794(3)	3576(4)	9737(5)	42.6(16)
H41a	1838(11)	4110(20)	10646(14)	62(2)
H41b	2112(19)	3657(11)	11653(15)	62(2)
H41c	2507(8)	4493(13)	11170(30)	62(2)
H28	4048(3)	1071(4)	8316(5)	50.3(18)
H9	804(3)	7499(4)	6531(5)	48.6(17)
H14	1637(3)	3449(4)	3717(5)	40.8(16)
H38a	1633(4)	479(14)	7410(30)	62(2)
H38b	991(19)	28(5)	7940(14)	62(2)
H38c	901(17)	565(12)	6909(15)	62(2)
H26	3104(3)	592(4)	5635(4)	43.3(16)
H34	1201(3)	302(4)	9729(5)	49.3(17)
H8	202(3)	6571(4)	7699(5)	47.6(17)
H23a	4003(5)	2840(4)	6330(30)	58(2)
H23b	4375(14)	3540(20)	5609(13)	58(2)
H23c	4520(10)	3560(19)	6790(20)	58(2)
H40a	3274(3)	2730(30)	11564(17)	65(2)
H40b	3652(10)	2670(20)	10506(14)	65(2)
H40c	3612(11)	3612(5)	11100(30)	65(2)
H20a	651(13)	4591(6)	5320(30)	64(2)

H20b	560(15)	3970(20)	4355(5)	64(2)
H20c	82(4)	3823(17)	5310(30)	64(2)
H37a	128(7)	982(17)	8890(30)	70(2)
H37b	177(6)	2059(16)	8850(30)	70(2)
H37c	-42(3)	1510(30)	7871(5)	70(2)
H19a	920(20)	2267(10)	4453(7)	64(2)
H19b	1143(15)	1872(5)	5520(20)	64(2)
H19c	382(7)	2203(11)	5350(30)	64(2)
H10	1834(3)	6946(4)	5847(5)	49.7(18)
H1	5454(12)	3240(50)	7470(90)	149(3)
H22a	4000(20)	5238(12)	5628(10)	81(3)
H22b	3500(8)	5566(4)	6500(40)	81(3)
H22c	4242(16)	5210(11)	6770(30)	81(3)

^{1}H, ^{13}C and ^{11}B NMR spectra

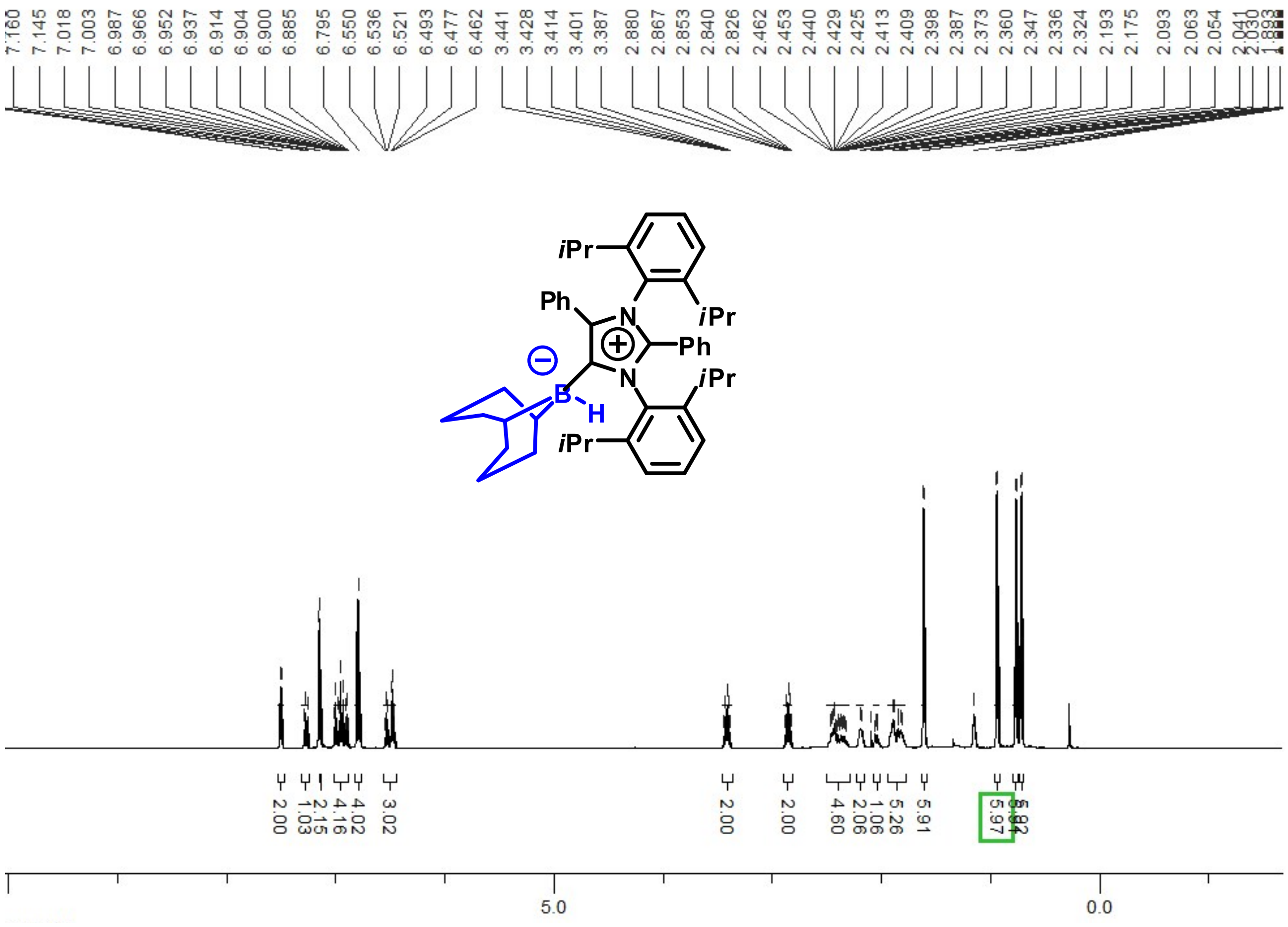

Fig. S5 ^{1}H NMR (C_6D_6, RT) spectrum of1,3-bis(2,6-diisopropylphenyl)-2,4-diphenylimidazolidine-9-borabicyclo[3.3.1]nonane (**2**).

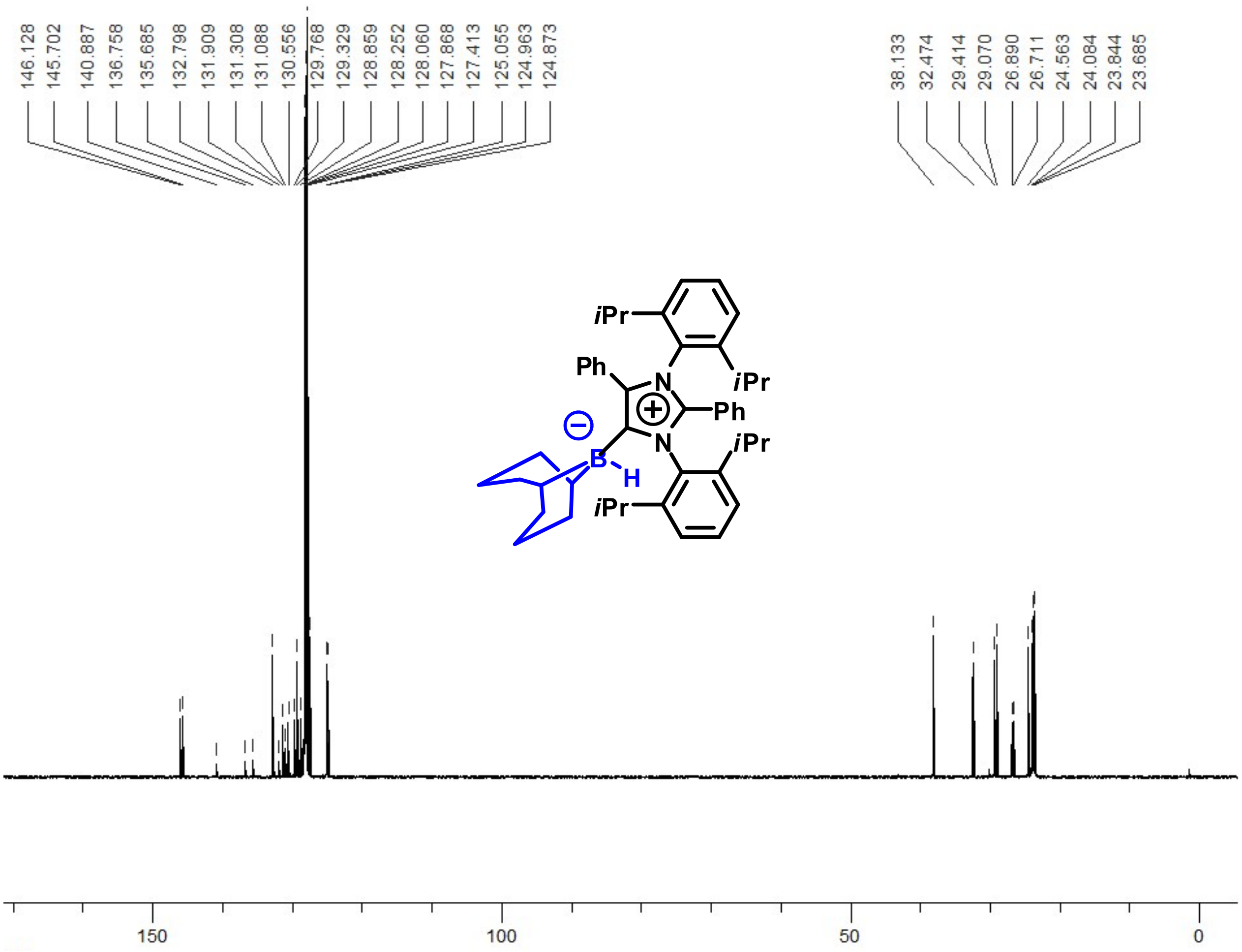

Fig. S6 ^{13}C NMR (C_6D_6, RT) spectrum of 1,3-bis(2,6-diisopropylphenyl)-2,4-diphenylimidazolidine-9-borabicyclo[3.3.1]nonane (**2**).

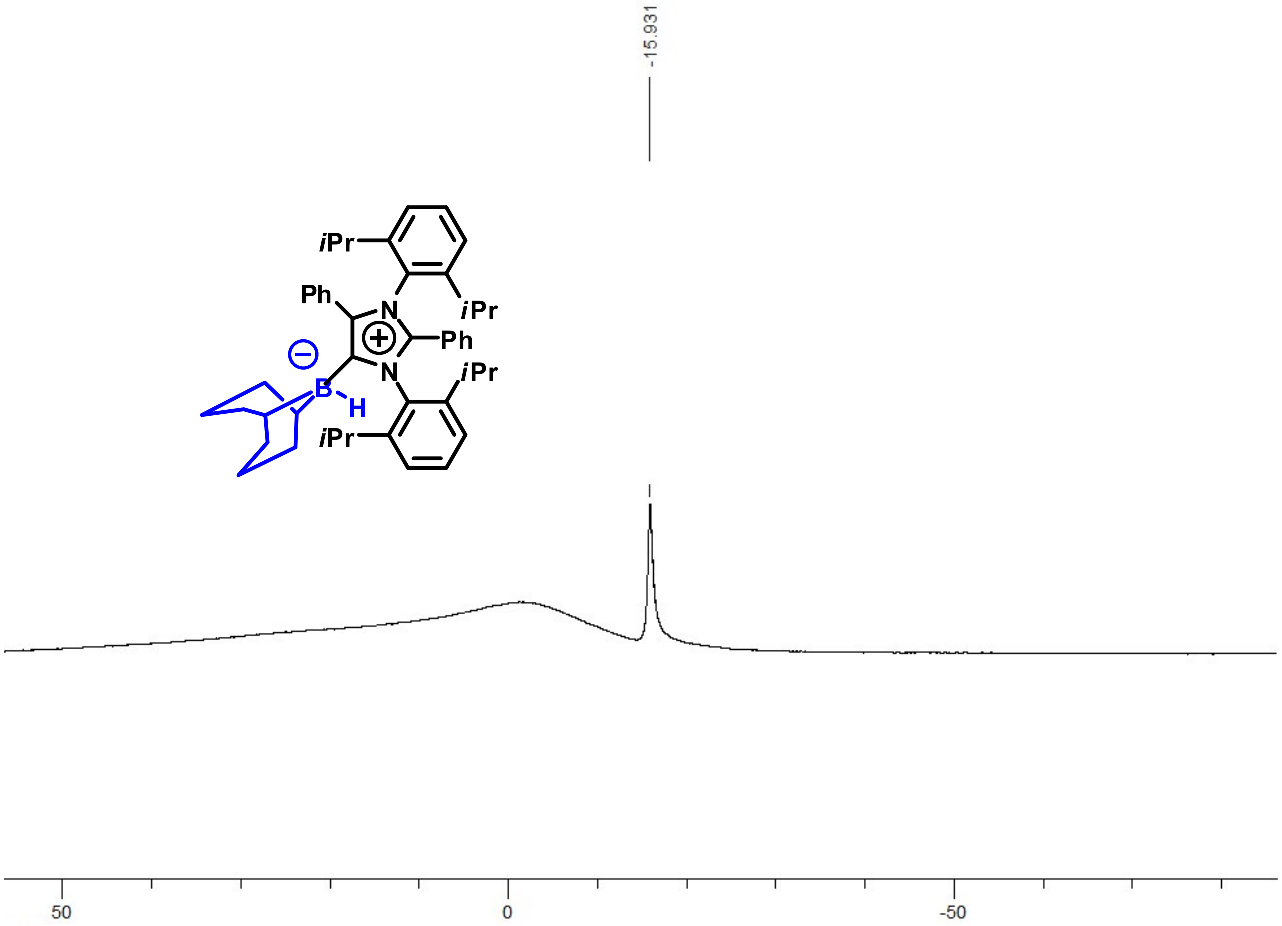

Fig. ***S7*** ^{11}B NMR (C_6D_6, RT) spectrum of 1,3-bis(2,6-diisopropylphenyl)-2,4-diphenylimidazolidine-9-borabicyclo[3.3.1]nonane (**2**).

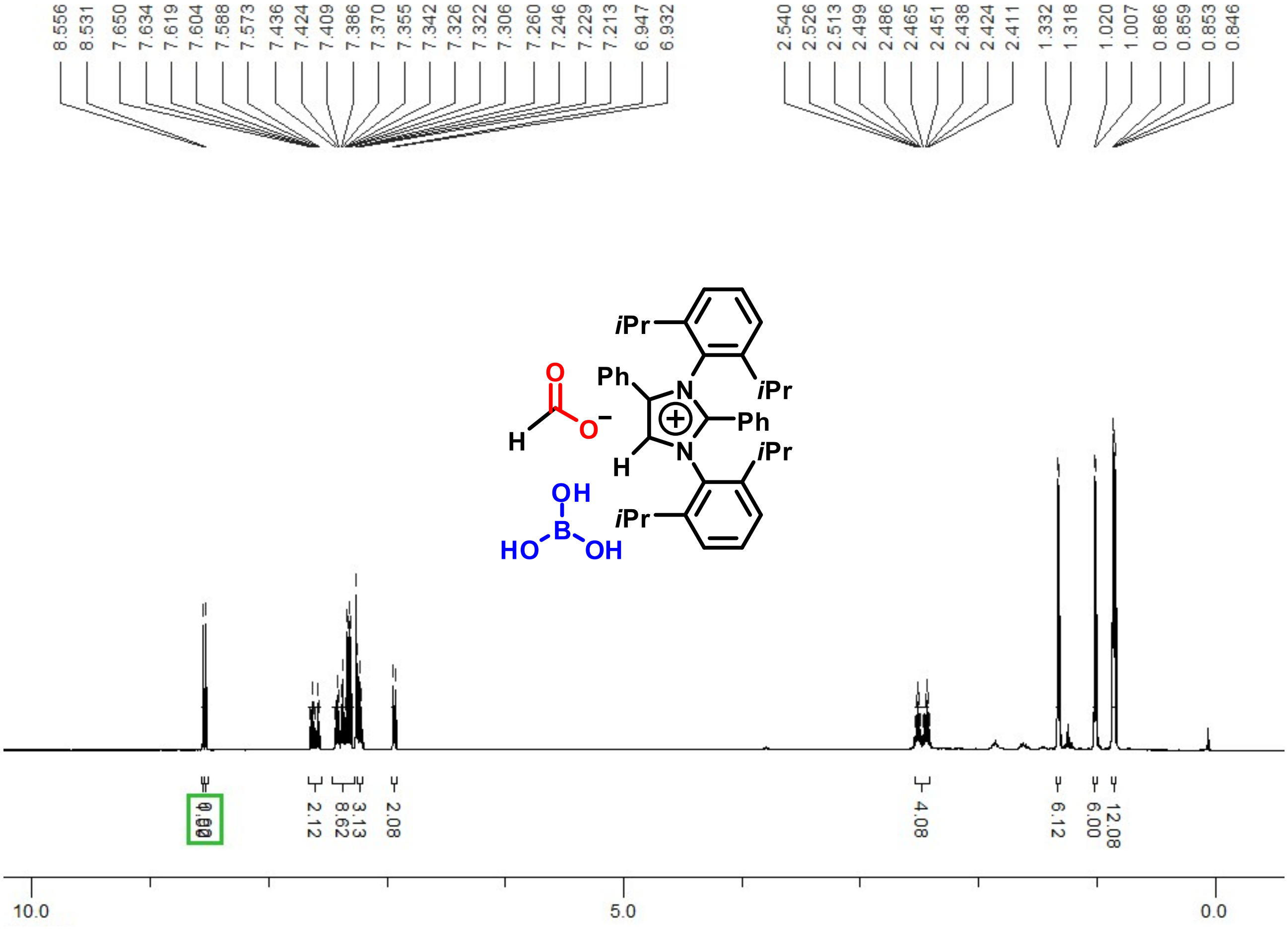

Fig. S8 ^{1}H NMR ($CDCl_3$, RT) spectrum of 1,3-bis(2,6-diisopropylphenyl)-2,4-diphenylimidazolidine formate boric acid (**3**).

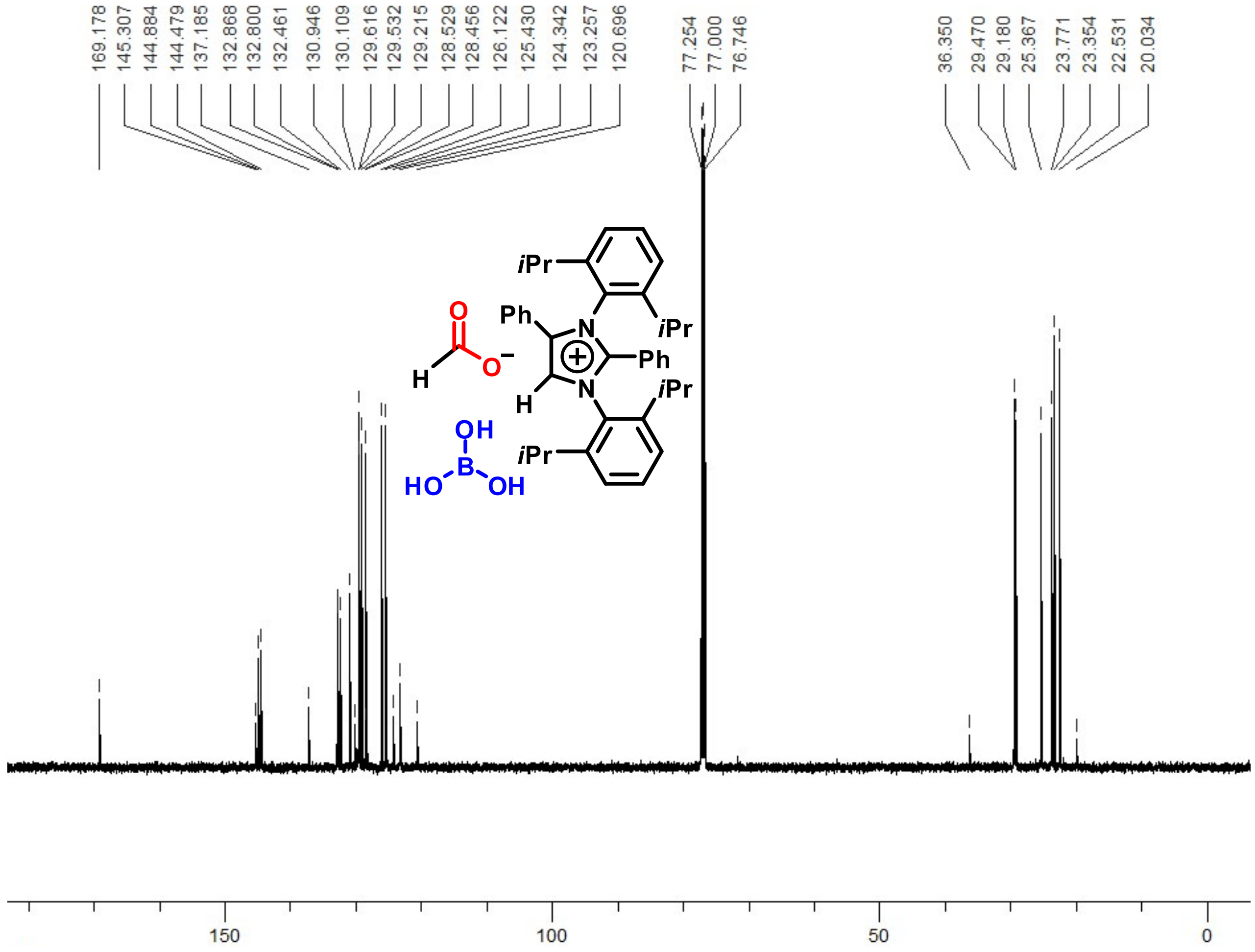

Fig. S9 ^{13}C NMR ($CDCl_3$, RT) spectrum of 1,3-bis(2,6-diisopropylphenyl)-2,4-diphenylimidazolidine formate boric acid (**3**).

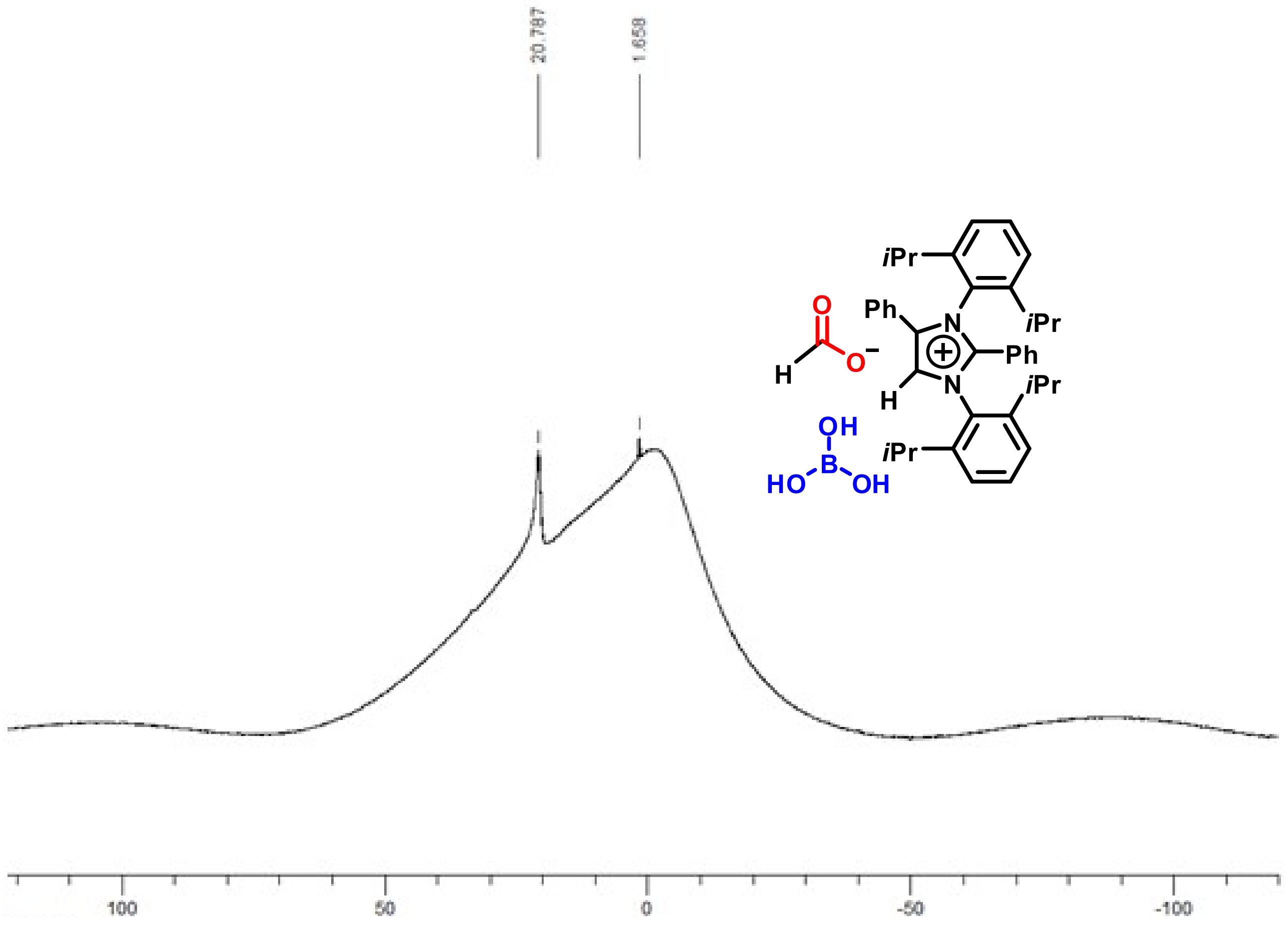

Fig. S10 ^{11}B NMR ($CDCl_3$, RT) spectrum of 1,3-bis(2,6-diisopropylphenyl)-2,4-diphenylimidazolidine formate boric acid (**3**).

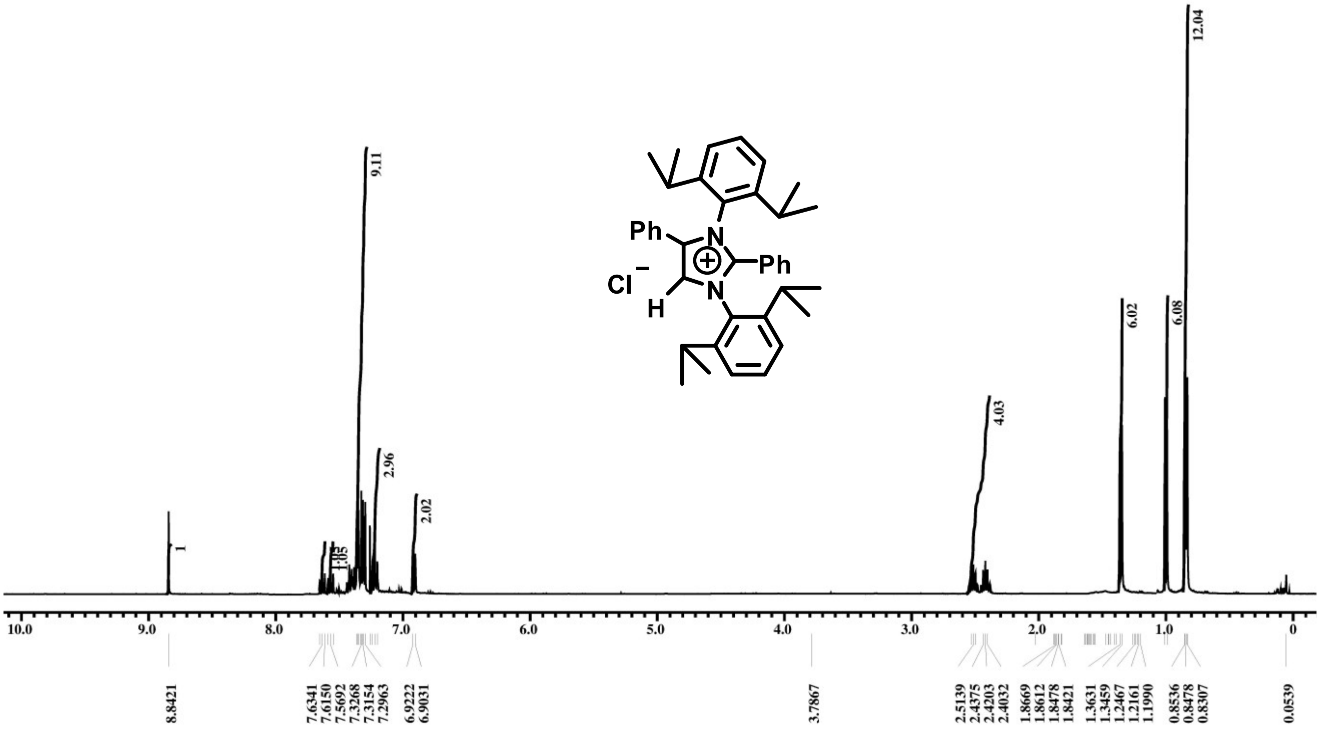

Fig. S11 ^{1}H NMR ($CDCl_3$, RT) spectrum of {1,3-bis(2,6-diisopropylphenyl)-2,4-diphenyl-imidazolium (Cl^-)} (**4**).

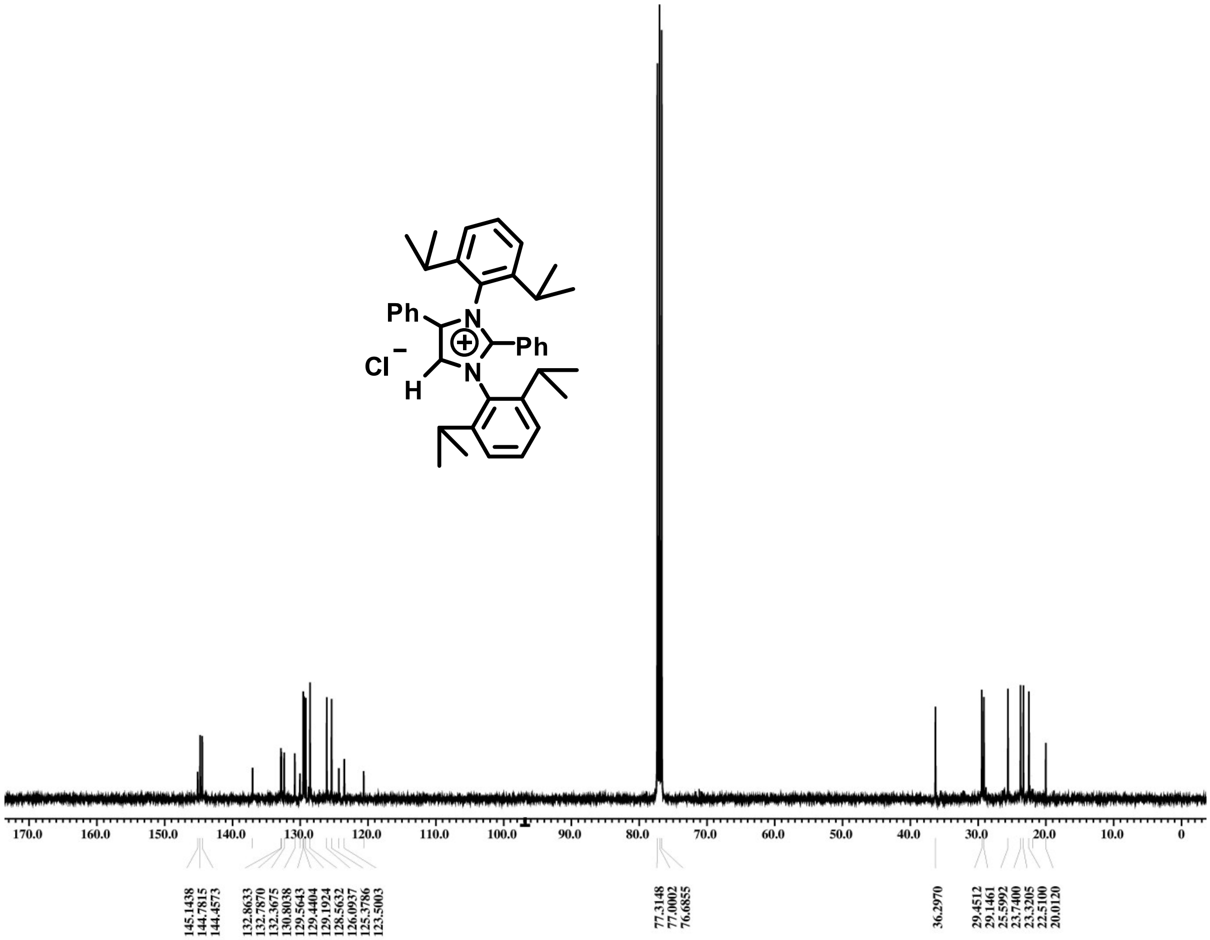

Fig. S12 ^{13}C NMR ($CDCl_3$, RT) spectrum of {1,3-bis(2,6-diisopropylphenyl)-2,4-diphenyl-imidazolium (Cl^-)} (**4**).

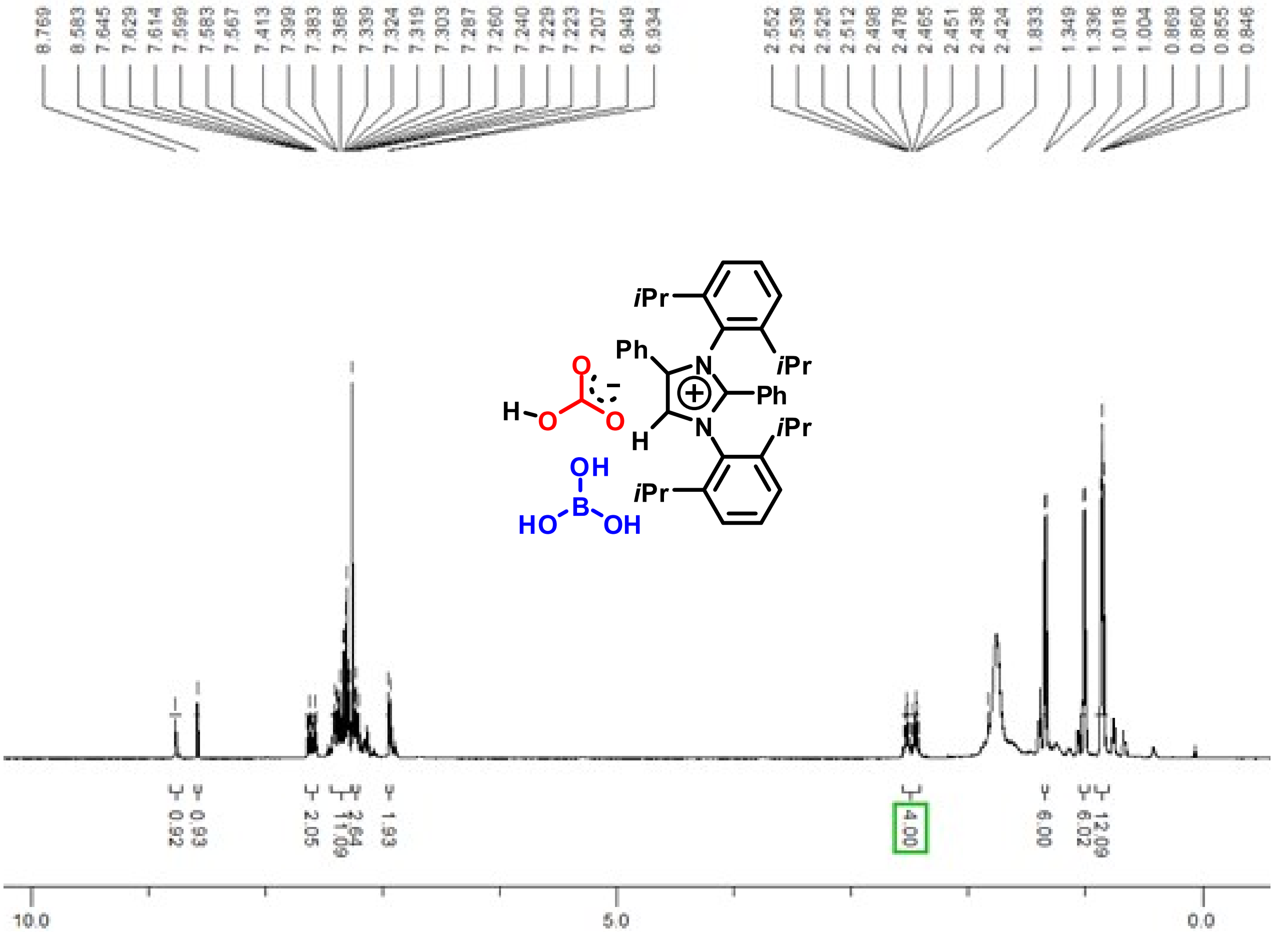

Fig. S13 ^{1}H NMR ($CDCl_3$, RT) spectrum of 1,3-bis(2,6-diisopropylphenyl)-2,4-diphenylimidazolidine bicarbonate boric acid} (**5**).

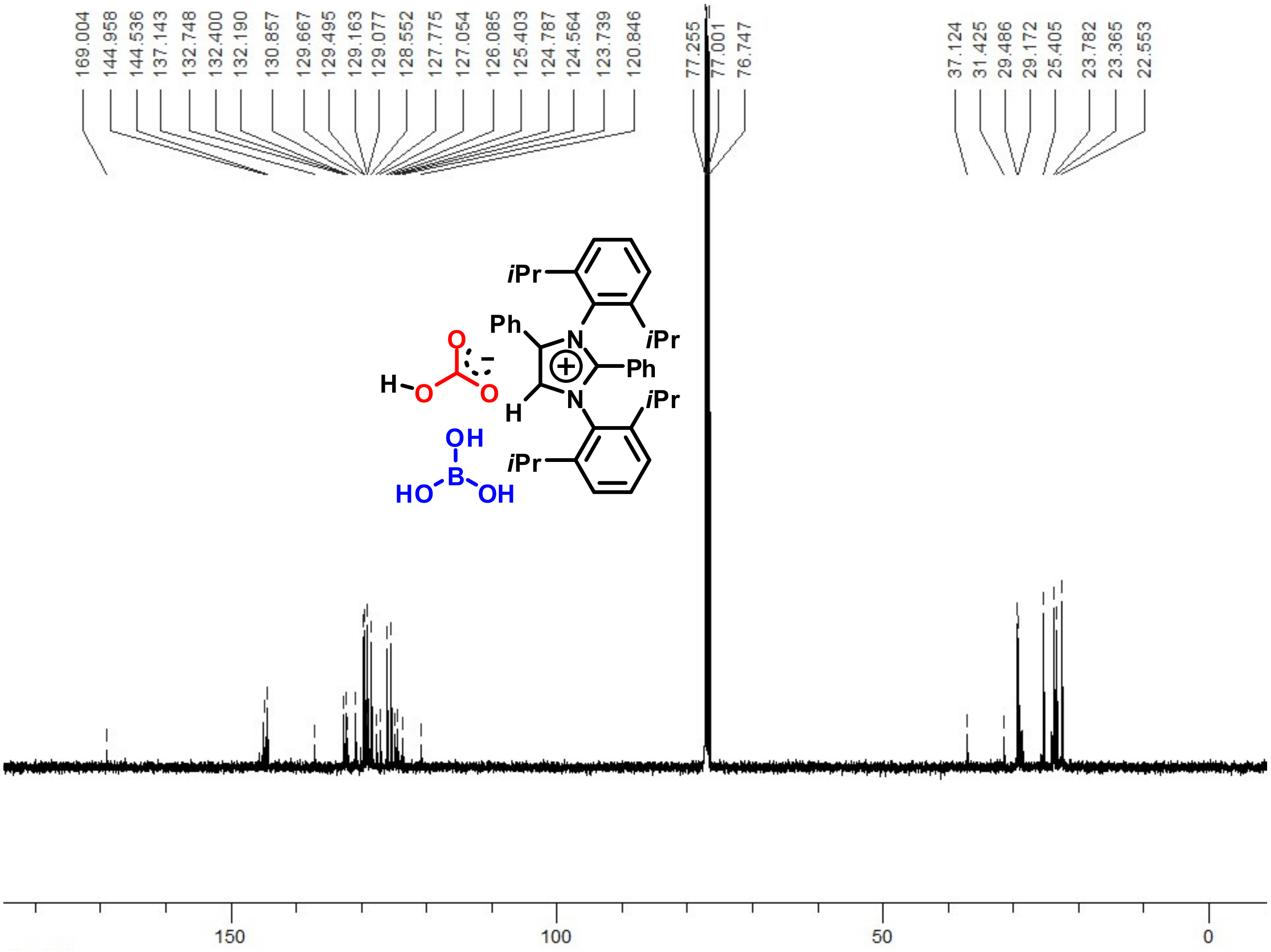

Fig. S14 ^{13}C NMR ($CDCl_3$, RT) spectrum of 1,3-bis(2,6-diisopropylphenyl)-2,4-diphenylimidazolidine bicarbonate boric acid} (**5**).

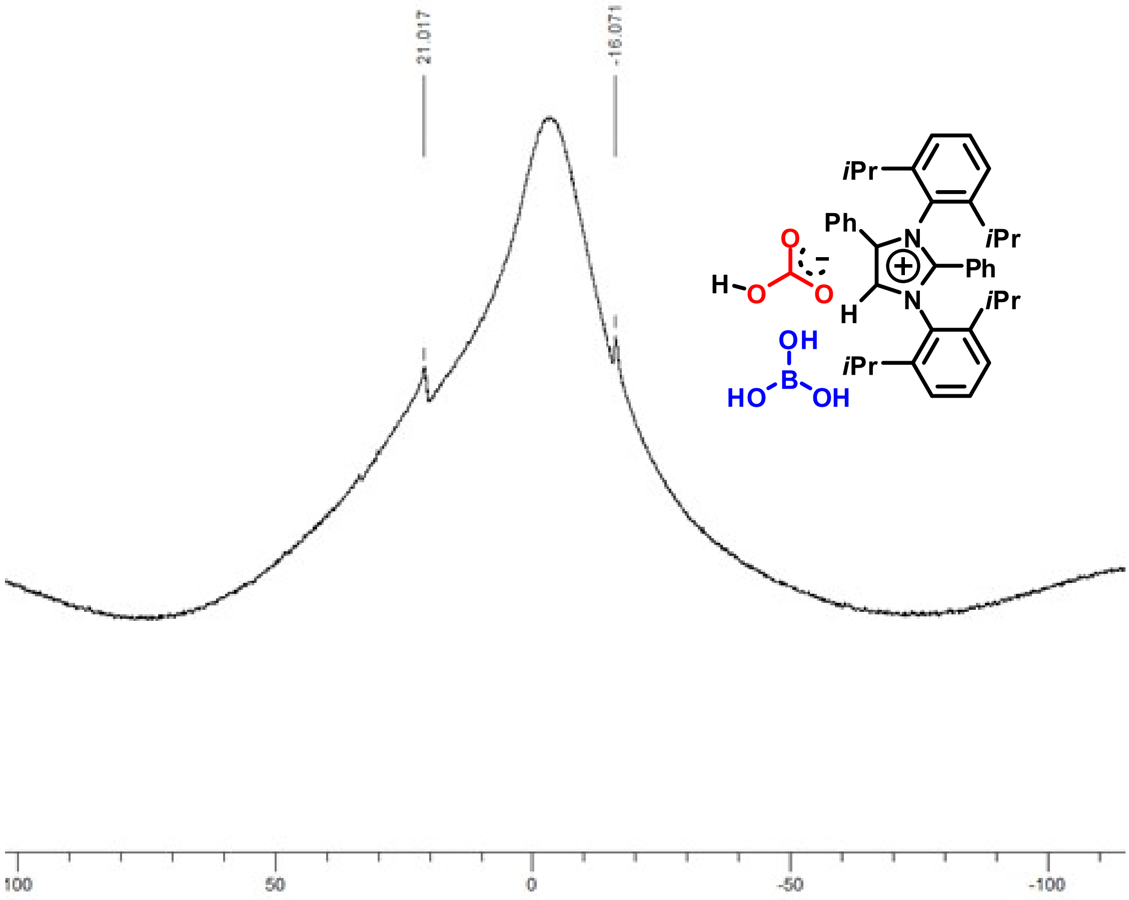

Fig. S15 ^{11}B NMR ($CDCl_3$, RT) spectrum of 1,3-bis(2,6-diisopropylphenyl)-2,4-diphenylimidazolidine bicarbonate boric acid} (**5**).

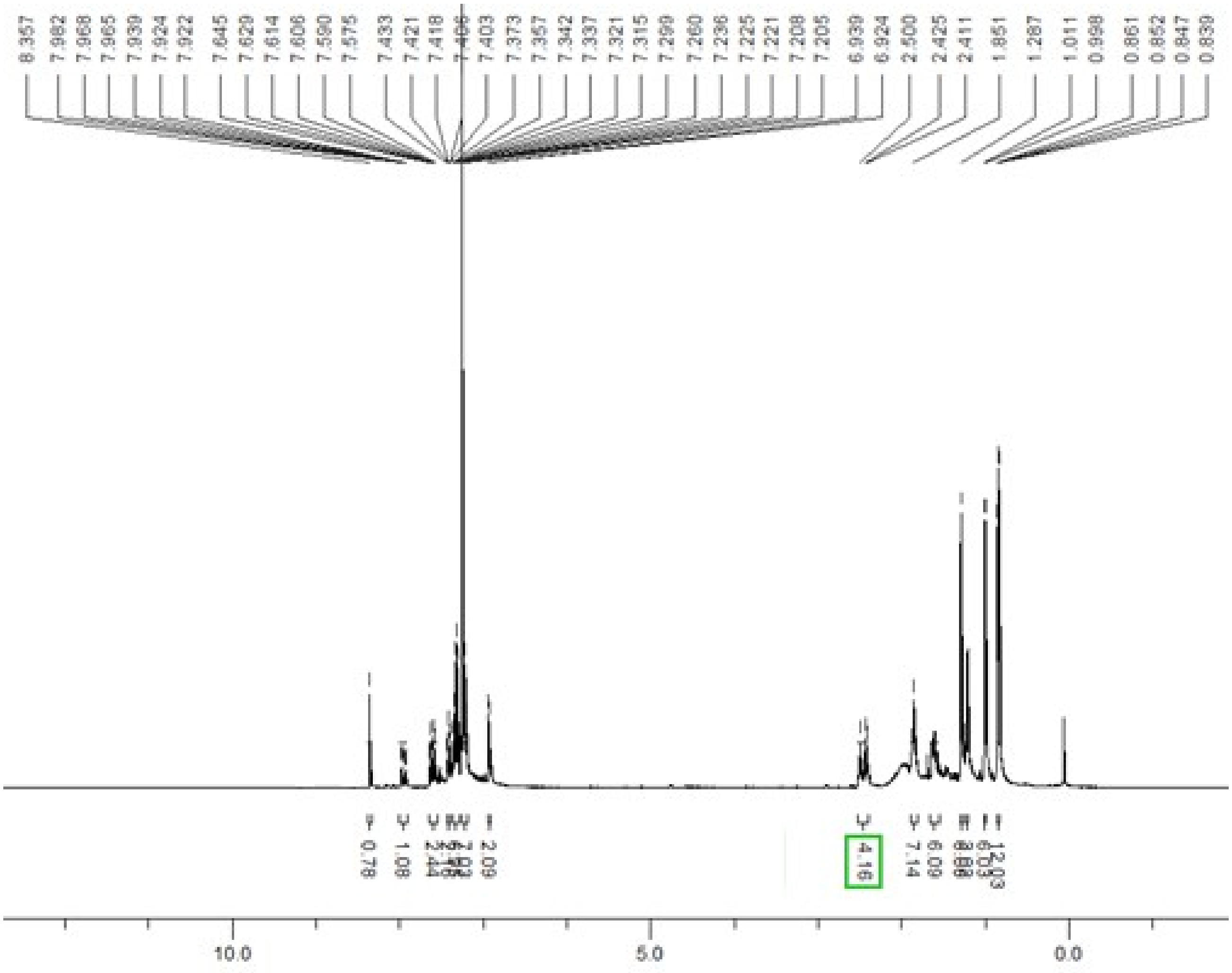

Fig. S16 ^{1}H NMR ($CDCl_3$, RT) spectrum of 1,3-bis(2,6-diisopropylphenyl)-2,4-diphenylimidazolidine formate boric acid (**3**) after heating at 150 °C for 12 h.

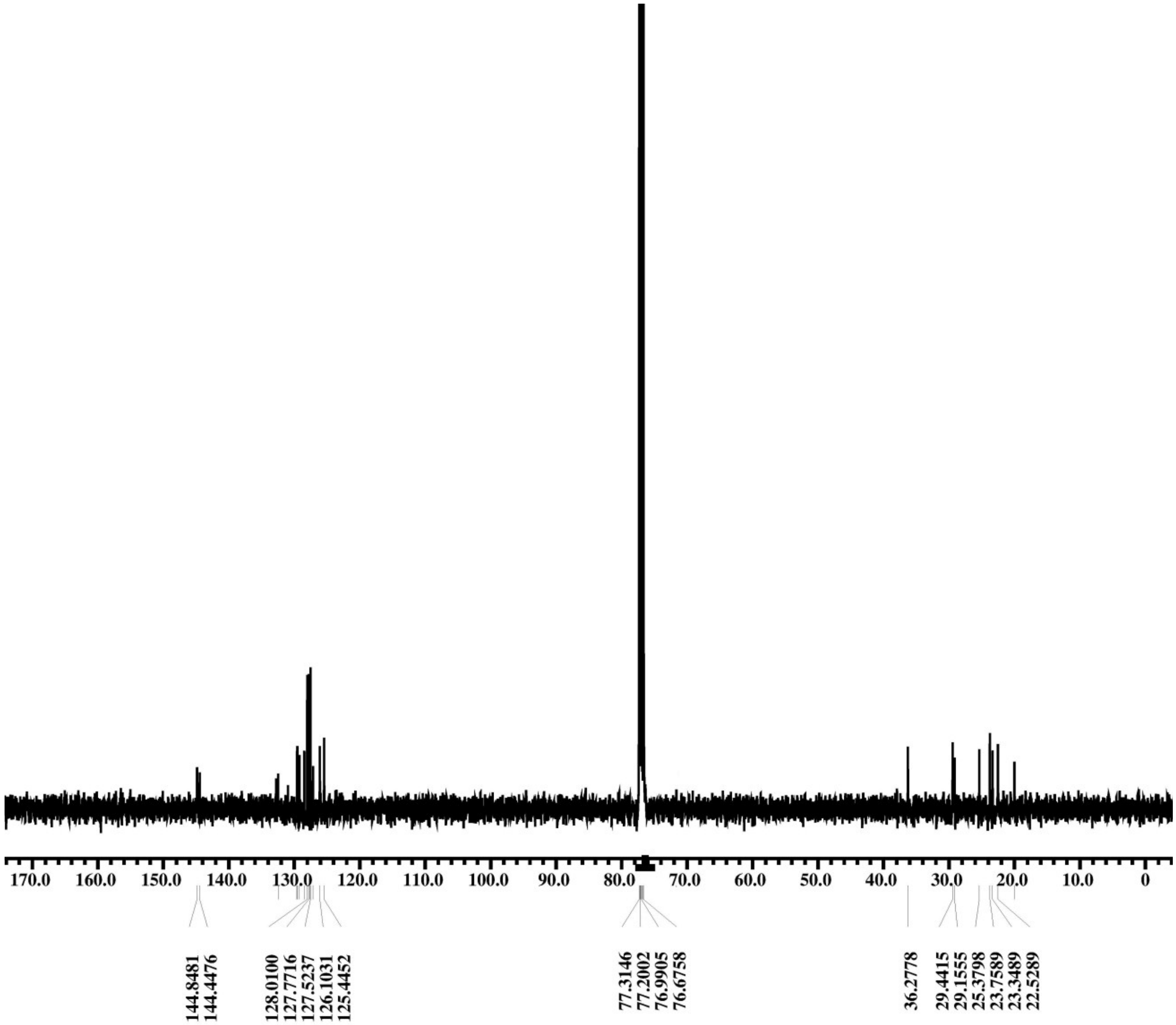

Fig. S17 ^{13}C NMR ($CDCl_3$, RT) spectrum of 1,3-bis(2,6-diisopropylphenyl)-2,4-diphenylimidazolidine formate boric acid (**3**) after heating at 150 °C for 12 h.

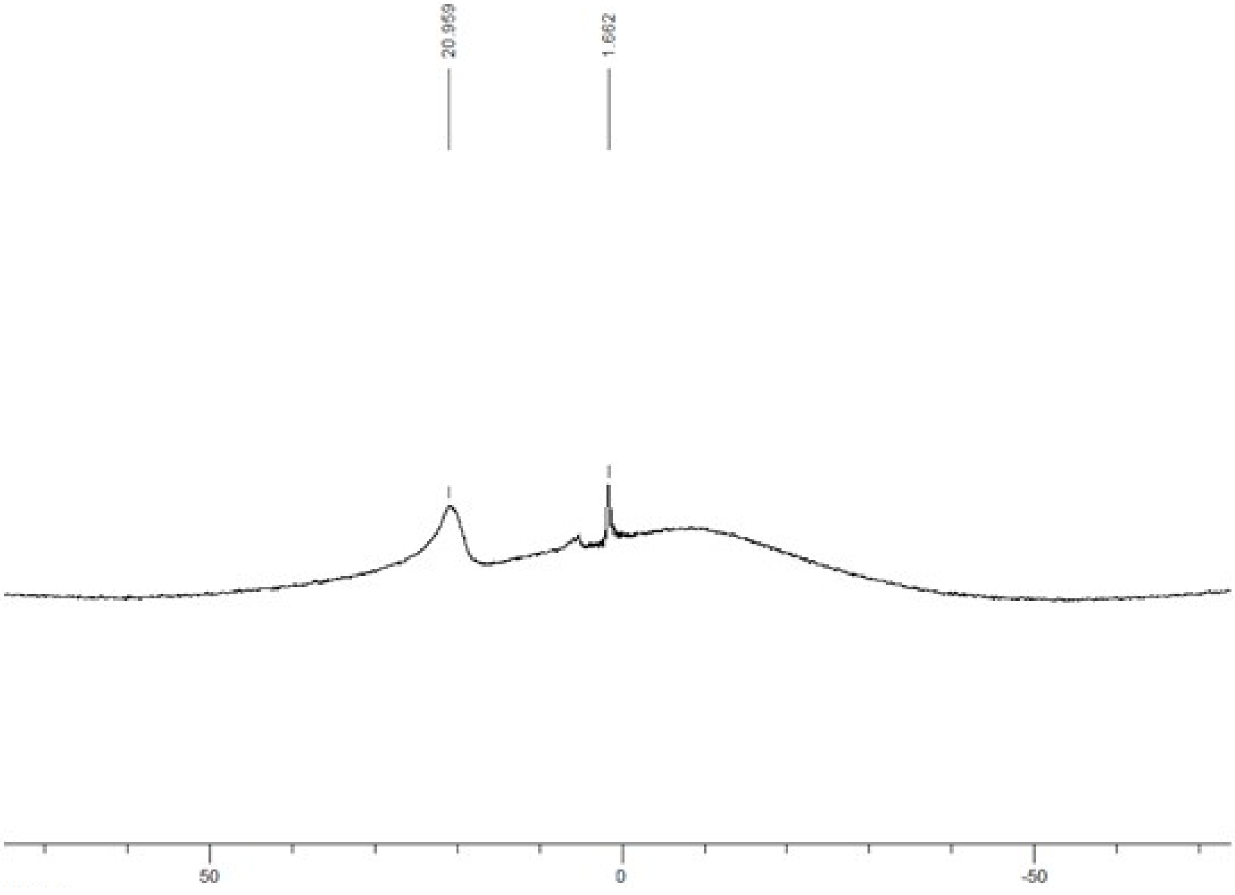

Fig. S18 ^{11}B NMR ($CDCl_3$, RT) spectrum of 1,3-bis(2,6-diisopropylphenyl)-2,4-diphenylimidazolidine formate boric acid (**3**) after heating at 150 ^{o}C for 12 h.

a

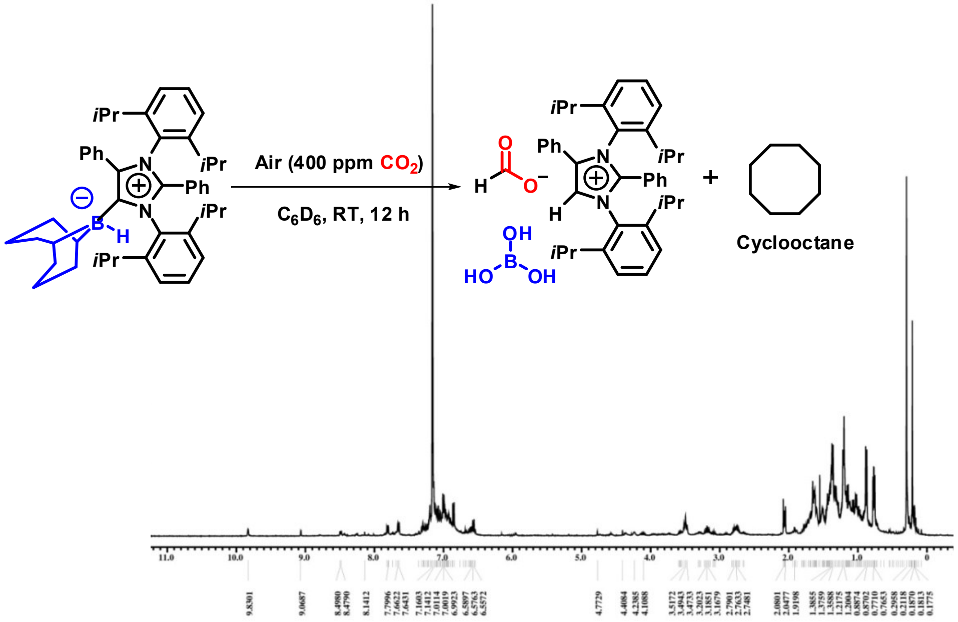

b

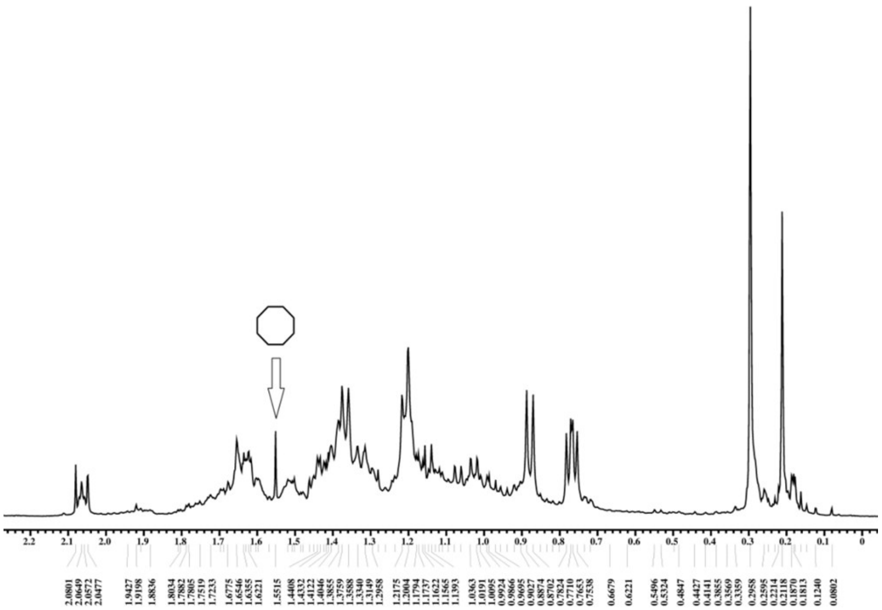

Fig. S19 Reaction mixture ^1H NMR (C_6D_6, RT) spectrum of 1,3-bis(2,6-diisopropylphenyl)-2,4-diphenylimidazolidine-9-borabicyclo[3.3.1]nonane (**2**) after exposing in air for 12 h. a) Full spectrum. b) Zoom spectrum of aliphatic region.

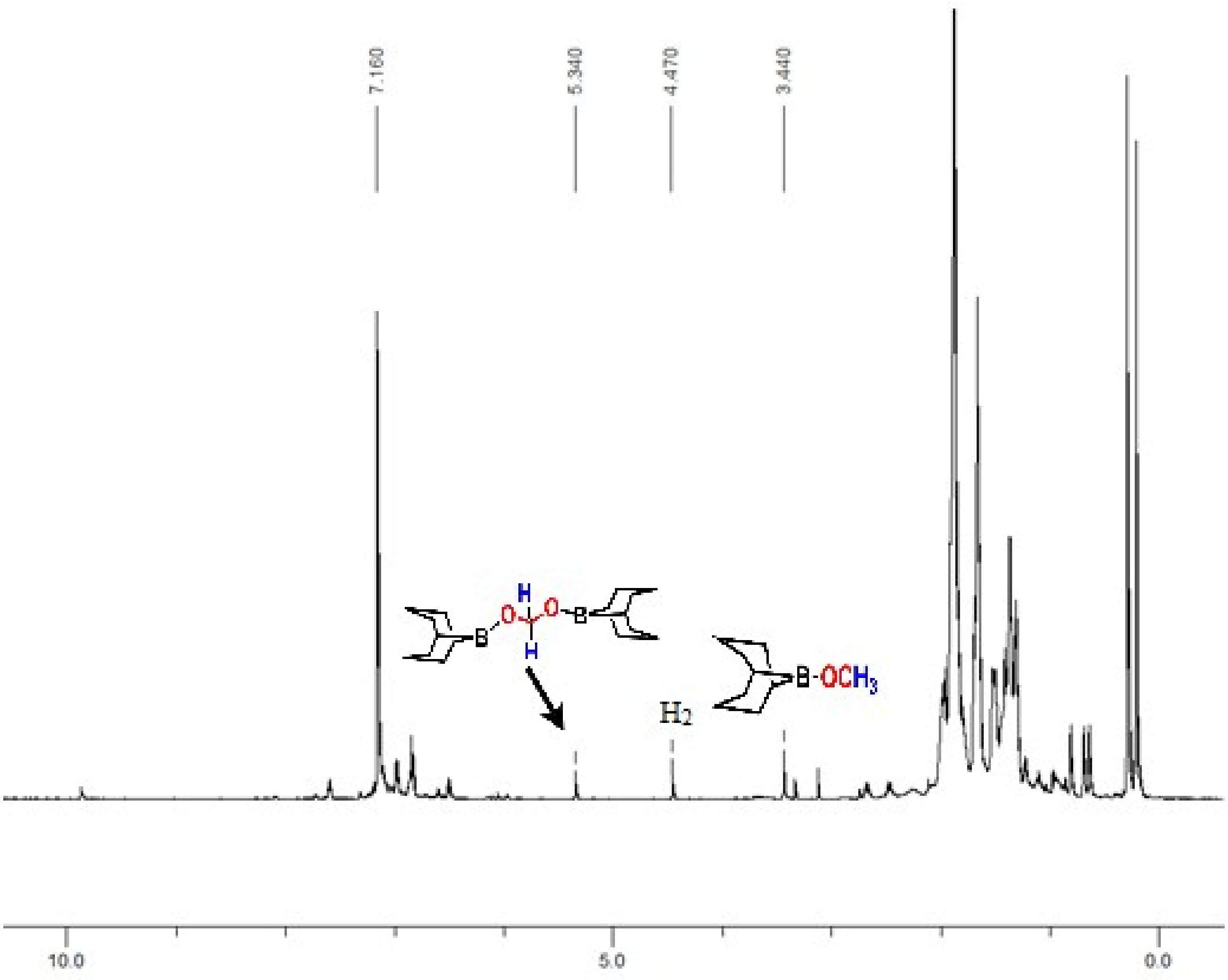

Fig. *S20* ^{1}H NMR (C_6D_6, RT) spectrum ofthe reaction mixture after 15 min (carbon dioxide reduction from air with compound **3** in presence of 10 equivalent 9-BBN at room temperature).

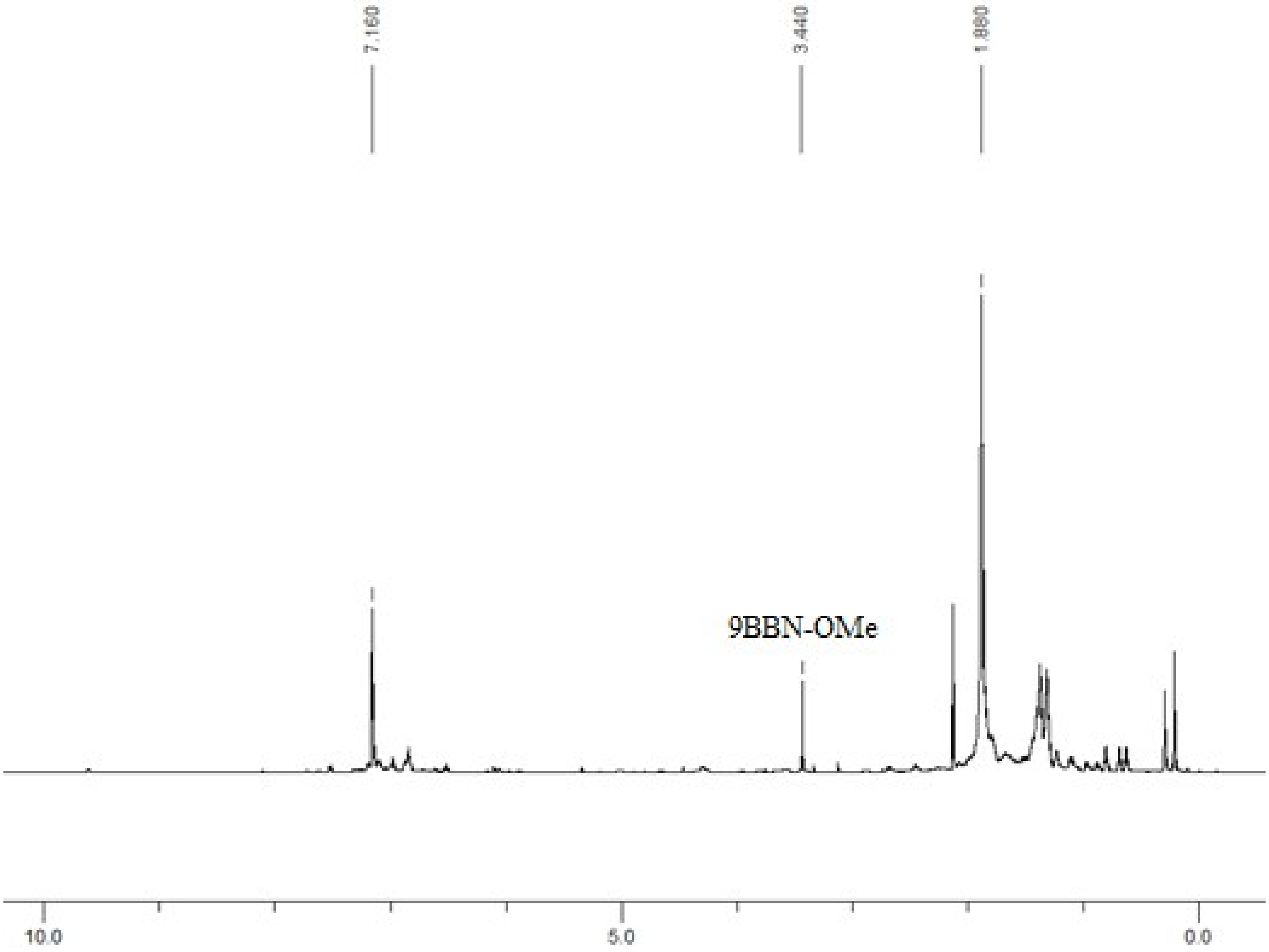

Fig. S21 ^1H NMR (C_6D_6, RT) spectrum of the reaction mixture after 6 h (carbon dioxide reduction from air with compound **3** in presence of 10 equivalents 9-BBN at room temperature).

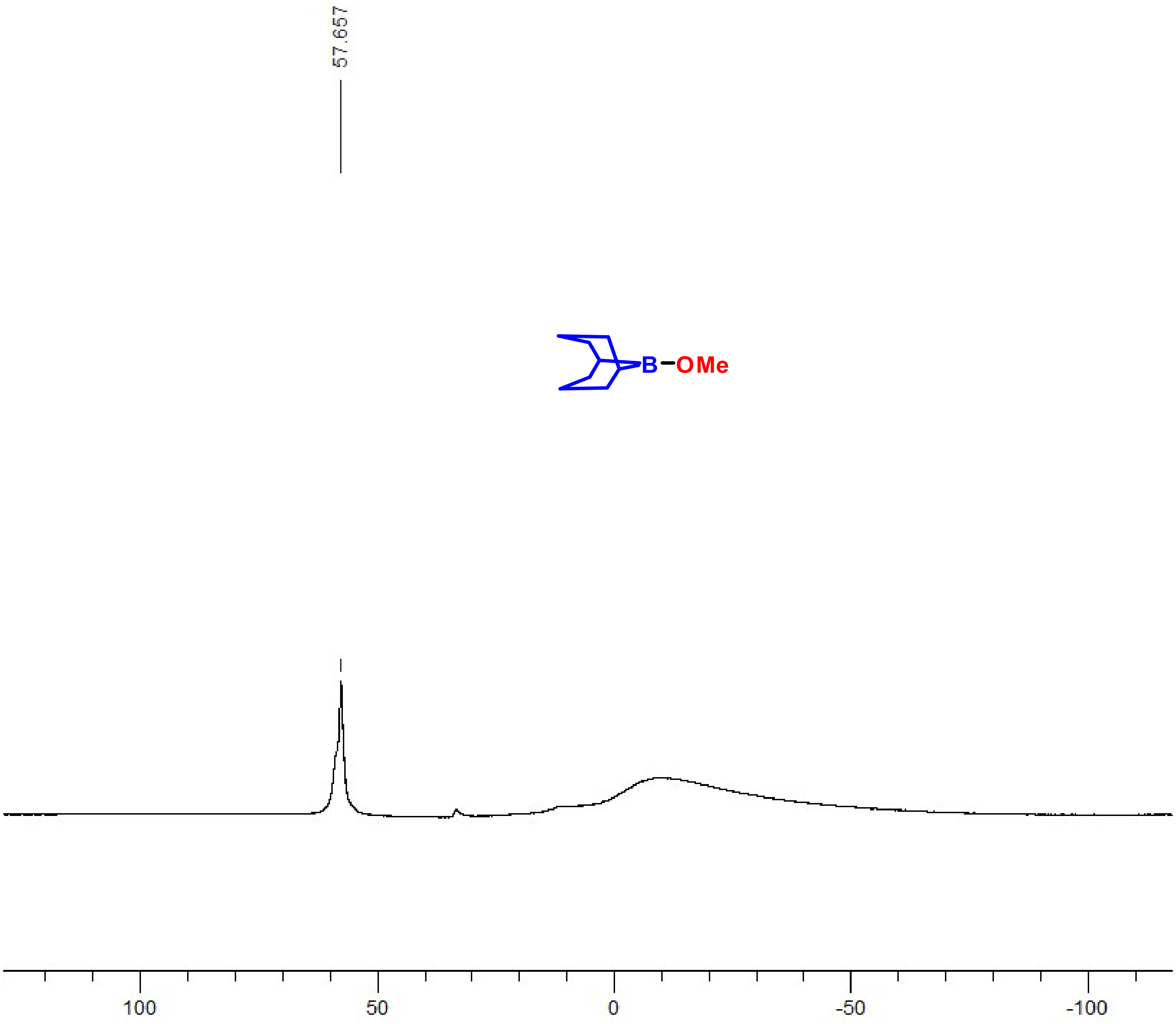

Fig. S22 ^{11}B NMR (C_6D_6, RT) spectrum of the reaction mixture after 6 h (carbon dioxide reduction from air with compound **3** in presence of 10 equivalents 9-BBN at room temperature).

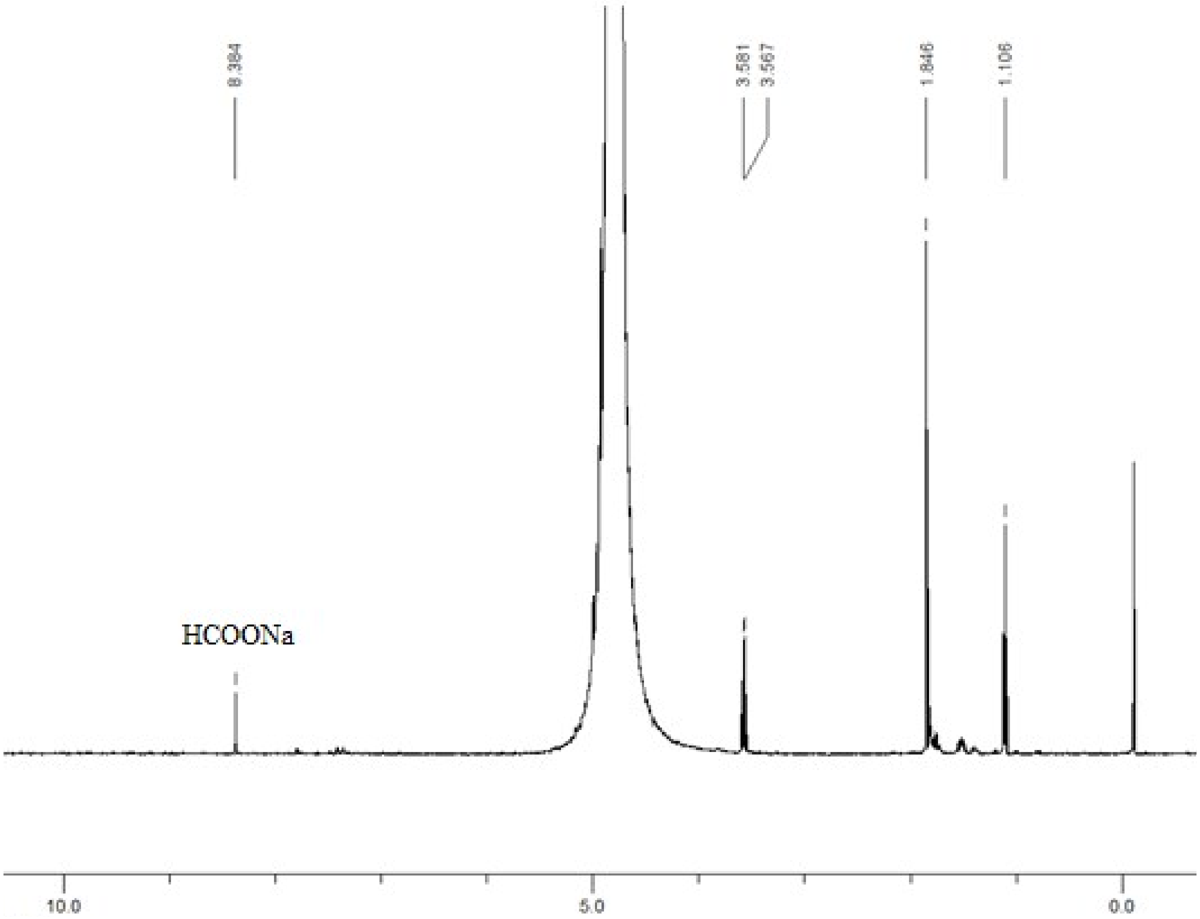

Fig. *S23* ^1H NMR (D_2O, RT) spectrum of the reaction mixture after 12 h (carbon dioxide reduction from air with compound **3** in presence of 2(M) NaOH solution at room temperature).

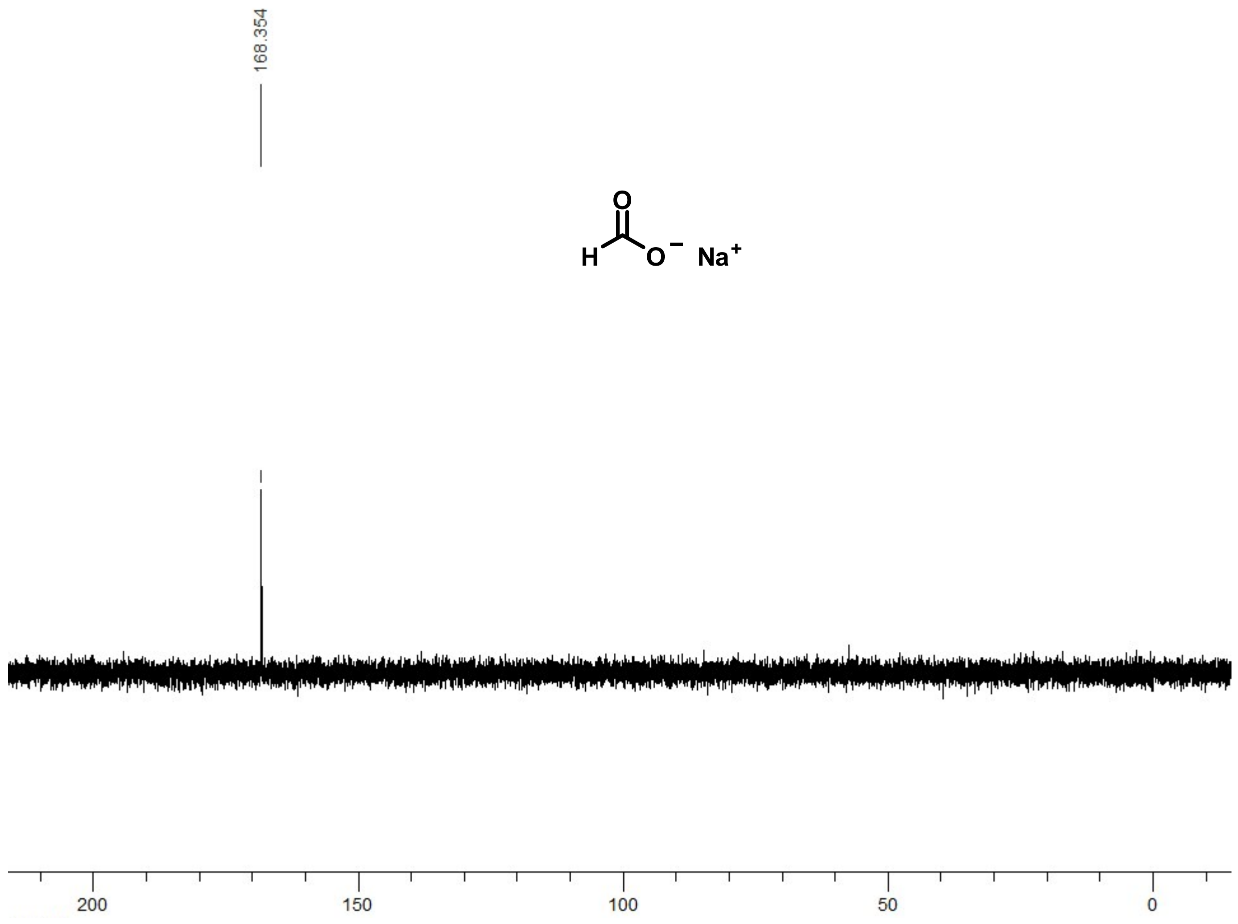

Fig. S24 ^{13}C NMR (D_2O, RT) spectrum of the reaction mixture after 12 h (carbon dioxide reduction from air with compound **3** in presence of 2(M) NaOH solution at room temperature).

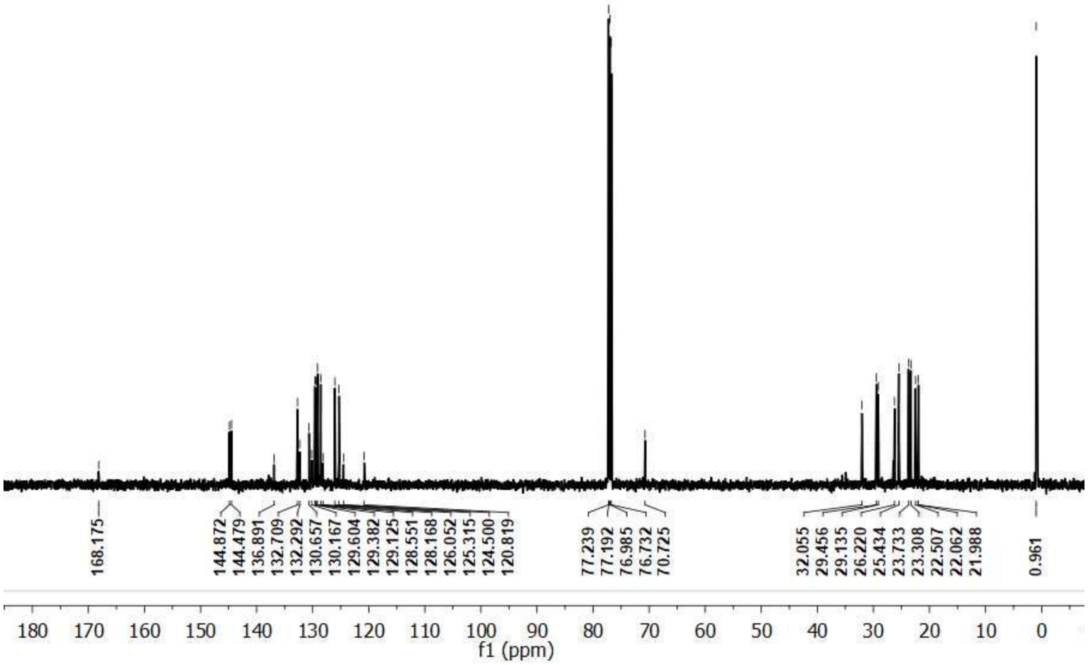

Fig. S25 ^{13}C NMR ($CDCl_3$, RT) spectrum of the reaction mixture after 12 h (reaction of compound **2** with dry air in toluene for 12 h at room temperature).

References

1. E. Aldeco-Perez, A. J. Rosenthal, B. Donnadieu, P. Parameswaran, G. Frenking and G. Bertrand, *Science*, 2009, **326**, 556–559.

2. P. Eisenberger, B. P. Bestvater, E. C. Keske and C. M. Crudden, *Angew. Chem. Int. Ed.,* 2015, **54**, 2467–2471.

3. Y. Zhao and D. G. Truhlar, *J. Chem. Theory Comput*., 2008, **4**, 1849–1868.

4. R. Bhattacharjee, A. Nijamudheen and A. Datta, *Org. Biomol. Chem*., 2015, **13**, 7412–7420.

5. A. V. Marenich, C. J. Cramer and D. G. Truhlar, *J. Phys. Chem. B,* 2009, **113**, 6378–6396.

6. M. J. Frisch, G. W. Trucks, H. B. Schlegel, G. E. Scuseria, M. A. Robb, J. R. Cheeseman, G. Scalmani, V. Barone, B. Mennucci, G. A. Petersson, H. Nakatsuji, M. Caricato, X. Li, H. P. Hratchian, A. F. Izmaylov, J. Bloino, G. Zheng, J. L. Sonnenberg, M. Hada, M. Ehara, K. Toyota, R. Fukuda, J. Hasegawa, M. Ishida, T. Nakajima, Y. Honda, O. Kitao, H. Nakai, T. Vreven, J. A. Montgomery, Jr. J. E. Peralta, F. Ogliaro, M. Bearpark, J. J. Heyd, E. Brothers, K. N. Kudin, V. N. Staroverov, R. Kobayashi, J. Normand, K. Raghavachari, A. Rendell, J. C. Burant, S. S. Iyengar, J. Tomasi, M. Cossi, N. Rega, J. M. Millam, M. Klene, J. E. Knox, J. B. Cross, V. Bakken, C. Adamo, J. Jaramillo, R. Gomperts, R. E. Stratmann, O. Yazyev, A. J. Austin, R. Cammi, C. Pomelli, J. W. Ochterski, R. L. Martin, K. Morokuma, V. G. Zakrzewski, G. A. Voth, P. Salvador, J. J. Dannenberg, S. Dapprich, A. D. Daniels, O. Farkas, J. B. Foresman, J. V. Ortiz, J. Cioslowski and D. J. Fox, Gaussian 09, revision A.1; Gaussian, Inc.: Wallingford, CT, 2009.

7. S. C. Sau, R. Bhattacharjee, P. K. Vardhanapu, G. Vijaykumar, A. Datta and S. K. Mandal, *Angew. Chem. Int. Ed*., 2016, **55**, 15147–15151; *Angew. Chem.,* 2016, **128**, 15371–15375.

8. A. D. Becke, *Phys. Rev. A,* 1988, **38**, 3098–3100.

9. C. Lee, W. Yang and R. G. Parr, *Phys. Rev. B,* 1988, **37**, 785–789.

10. S. Dapprich, I. Komáromi, K. S. Byun, K. Morokuma and M. J. Frisch, *J. Mol. Struct.: THEOCHEM.,* 1999, **462**, 1–21.

11. M. Svensson, S. Humbel, R. D. J. Froese, T. Matsubara, S. Sieber and K. Morokuma, *J. Phys. Chem.,* 1996, **100**, 19357–19363.

12. R. Bhattacharjee, A. Nijamudheen, S. Karmakar and A. Datta, *Inorg. Chem.,* 2016, **55**, 3023–3029.

13. M. J. S. Dewar, E. G. Zoebisch, E. F. Healy and J. P. Stewart, *J. Am. Chem. Soc.,* 1985, **107**, 3902–3909.

14. M.-A. Courtemanche, M.-A. Légaré, L. Maron and F.-G. Fontaine, *J. Am. Chem. Soc.,* 2014, **136**, 10708–10717.

15. O. V. Dolomanov, L. J. Bourhis, R. J. Gildea, J. A. K. Howard and H. Puschmann, *J. Appl. Cryst.,* 2009, **42**, 339–341.

16. L. Palatinus and G. Chapuis, *J. Appl. Cryst.,* 2007, **40**, 786–790.

17. G. M. Sheldrick, *Acta Cryst.,* 2008, **A64**, 112–122.

18. G. M. Sheldrick, SHELXS97 and SHELXL97. Program for crystal structure solution and refinement, University of Göttingen, Germany, 1997.
